# Natural Killer Receptor 1 Dampens the Development of Allergic Eosinophilic Airway Inflammation

**DOI:** 10.1371/journal.pone.0160779

**Published:** 2016-08-31

**Authors:** Shirin Elhaik Goldman, Itay Moshkovits, Avishai Shemesh, Ayelet Filiba, Yevgeny Tsirulsky, Elena Vronov, Marilou Shagan, Ron N. Apte, D aniel Benharroch, Danielle Karo-Atar, Ron Dagan, Ariel Munitz, Yaffa Mizrachi Nebenzahl, Angel Porgador

**Affiliations:** 1 The Shraga Segal Department of Microbiology, Immunology and Genetics, Ben Gurion University of the Negev, Beer Sheva 84105, Israel; 2 Department of Clinical Microbiology and Immunology, Sackler school of medicine, Tel-Aviv University, Tel Aviv, Israel; 3 Soroka University Medical Center, Department of Pathology, Bear Sheva, Israel; 4 Faculty of Health Science, Ben Gurion University of the Negev, Beer Sheva 84105, Israel; 5 National Institute of Biotechnology in the Negev, Ben-Gurion University of the Negev, Beer Sheva, Israel; Mie University Graduate School of Medicine, JAPAN

## Abstract

The function of NCR1 was studied in a model of experimental asthma, classified as a type 1 hypersensitivity reaction, in mice. IgE levels were significantly increased in the serum of OVA immunized NCR1 deficient (*NCR1*^*gfp/gfp*^) mice in comparison to OVA immunized wild type *(NCR1*^*+/+*^) and adjuvant immunized mice. Histological analysis of OVA immunized *NCR1*^*gfp/gfp*^ mice revealed no preservation of the lung structure and overwhelming peribronchial and perivascular granulocytes together with mononuclear cells infiltration. OVA immunized *NCR*^*+/+*^ mice demonstrated preserved lung structure and peribronchial and perivascular immune cell infiltration to a lower extent than that in *NCR1*^*gfp/gfp*^ mice. Adjuvant immunized mice demonstrated lung structure preservation and no immune cell infiltration. OVA immunization caused an increase in PAS production independently of NCR1 presence. Bronchoalveolar lavage (BAL) revealed NCR1 dependent decreased percentages of eosinophils and increased percentages of lymphocytes and macrophages following OVA immunization. In the OVA immunized *NCR1*^*gfp/*gfp^ mice the protein levels of eosinophils’ (CCL24) and Th2 CD4^+^ T-cells’ chemoattractants (CCL17, and CCL24) in the BAL are increased in comparison with OVA immunized *NCR*^*+/+*^ mice. In the presence of NCR1, OVA immunization caused an increase in NK cells numbers and decreased NCR1 ligand expression on CD11c^+^GR1^+^ cells and decreased NCR1 mRNA expression in the BAL. OVA immunization resulted in significantly increased IL-13, IL-4 and CCL17 mRNA expression in *NCR1*^*+/+*^ and *NCR1*^*gfp/*gfp^ mice. IL-17 and TNFα expression increased only in OVA-immunized *NCR1*^*+/+*^mice. IL-6 mRNA increased only in OVA immunized *NCR1*^*gfp/gfp*^ mice. Collectively, it is demonstrated that NCR1 dampens allergic eosinophilic airway inflammation.

## Introduction

Allergic asthma, classified as type 1 hypersensitivity reaction, is a chronic inflammatory disease of the airways characterized by reversible airflow obstruction, bronchial hyper-responsiveness, airway inflammation and production of allergy specific immunoglobulin E (IgE) [1, 2]. Studies of airway inflammation in the lungs of asthmatic individuals have revealed the accumulation of a large number of inflammatory cells (predominantly eosinophils), increased mucus production and sub-mucosal mucus glands hyperplasia/metaplasia, epithelial shedding, and smooth muscle cell hypertrophy leading to structural changes that in turn exacerbate the hyper-responsiveness observed in this disease. Research in the asthma field has provided rationale for the development of multiple therapeutic agents that interfere with specific inflammatory pathways. However, our understanding of asthma is limited as recent genome searches have revealed at least 19 genes that contribute to asthma susceptibility and microarray studies of asthmatic tissue demonstrated increased expression of 291 genes that were commonly involved in murine disease pathogenesis independent of the mode of disease induction [3, 4]. Therefore, a central issue still being studied is identification of fundamental molecules / pathways that govern the processes underlying inflammation in asthma.

In predisposed individuals, initial exposure(s) of professional antigen-presenting cells (APCs) to an allergen leads mainly to the activation of allergen-specific T helper 2 (Th2) cells and IgE synthesis, which is known as allergic sensitization [2]. IgE-sensitized mast cells release both pre-formed and newly synthesized mediators, which promote vascular permeability, smooth-muscle contraction, and mucus production. Chemokines released by mast cells direct the recruitment of inflammatory cells that contribute to the late allergic response. This stage of allergic response is characterized by an influx of eosinophils (via CCL24 and CCL11, which bind to CCR3) and Th2 cells (via CCL17 and CCL22, which bind CCR4). The eosinophils release a large number of pro-inflammatory mediators and toxic granules, which cause bronchoconstriction and damage to the epithelial cell layer [2, 5]. The polarized immunity toward Th2 phenotype in allergic respiratory disease involves the secretion of IL-4, IL-5, IL-9, and IL-13 that favors humoral antibody production (primarily IgE) [1, 5]. IL-4 is vital for the regulation of growth, differentiation, activation, and functions of B cells and also induces isotype switching leading to IgE production. Importantly, IL-13 is a potent Th2 proinflammatory cytokine and its neutralization prevents airway hyper-responsiveness (AHR) in animal models. The importance of Th2 cytokines to the allergic induced AHR is inconclusive since neutralization of IL-4 / IL-13 failed to improve the allergic status in human clinical trials [6].

The earliest contact between an antigen and the innate immune cells is thought to direct the subsequent antigen-specific T-cell response. Thus, cells of the innate immune system, such as NK cells, NKT cells, and γδT cells might regulate the development of allergic airway disease. The ability of NK cells to produce cytokines and chemokines in response to direct or indirect stimuli helps them regulate multiple immune responses. NK cells can rapidly produce IFNγ along with myriad of cytokine and chemokines that attract DCs and macrophages to the area of inflammation. This co-localization of DCs and NK cells results in a cross talk, which can influence the maturation and activation of both cells. Additionally, NK cells demonstrate contact-dependent co-stimulation. NK cells express several co-stimulatory ligands allowing them to provide direct co-stimulation to T and B cells [7].

Recent studies suggest contradictory roles for NK cells in immune modulation of allergy [8]. It has been demonstrated that patients with asthma show increased numbers of NK cells in peripheral blood than in normal healthy blood donors [9–11]. Specifically, increased NK cells and high levels of IL-4^+^CD56^+^ NK2 cells in PBMCs of allergic rhinitis patients were shown [12]. In addition, in the peripheral blood CD56^+^CD16^+/–^NK cells were able to respond to DCs by proliferation and production of IFNγ and were found to be significantly reduced in patients with allergic rhinitis and intermittent asthma [7, 12, 13]. Similar discrepancies were described in the animal models for allergic inflammation [14–16]. Together these studies suggest that NK cells accelerate allergic inflammation. In contrast, human NK cells were shown to induce neutrophil and eosinophil cells apoptosis, suggesting a role of NK cells in resolving allergic inflammation [17, 18]. Thus NK cells are a double edge sword and in contemplating NK cells as therapeutic targets, they should be approached cautiously and additional studies to understand the involvement of NK cells in allergic inflammation are needed.

Natural killer (NK) cell activation is controlled by the integration of signals from activation and inhibitory receptors interactions [19–21]. Upon ligation of activating receptors with membrane-bound molecules on surrounding tissues, NK cells would undergo blastogenesis, cytokine production, acquire cytotoxicity ability and migration abilities. However, colligation of activating receptors with inhibitory receptors results in a dominant inhibitory effect that down regulates signals initiated via the activating pathways [22]. Whenever the inhibitory receptors recognize different alleles of human leukocyte antigen (HLA) class I molecules expressed on normal cells they transfer an inhibitory signal to the cell. In human NK cells both HLA-specific and non HLA-specific inhibitory receptors have been identified [22]. The inhibitory receptors specific for HLA class I belong either to killer cell Ig-like receptor (KIR) or to killer cell lectin-like receptors (KLR) [23]. This inhibitory signal prevents the killing of normal cells and limits the production of inflammatory cytokines including IFNγ, TNFα and GM-CSF from NK cells [22]. The loss in expression of class I molecules is usually a consequence of tumor transformation or viral infection which reduces the signaling of the inhibitory receptors [20, 24–26]. The loss of inhibitory signal together with recognition of non-MHC ligands expressed on abnormal and normal cells will trigger NK cells activation. The main activating receptors are NKG2D and natural cytotoxicity receptors (NCRs, including NKp30, NKp44 and NKp46) that trigger the NK-target cell lysis through direct contact with the ligand. All of the NCRs are transmembrane proteins, members of the Ig-Superfamily [22]. Knocking down of NCR1 in NK cells reduce NCR1-mediated activity of NK cells, including NCR1-mediated lysis of target cells, but does not affect NK activity mediated by other NK receptors as NKG2D [27, 28]. Currently we analyzed the contribution of NCR1 to the allergic hypersensitivity response and found that its involvement in the resolution of the allergic hypersensitivity response.

## Materials and Methods

### Mice and ethic statement

C57BL/6 mouse strain *NCR1*^*gfp/gfp*^ (kindly provided by O. Mandelboim) and *NCR1*^*+/+*^ wild type littermates were used. In these mice, as described previously [29], the gene encoding the NCR1 receptor (*NCR1*) was replaced with a green fluorescent protein (GFP) reporter cassette. The animals used were 8 to 12 weeks old, unless otherwise stated. Age- and sex-matched animals served as controls.

This study was carried out in strict accordance with the recommendations in the Guide for the Care and Use of Laboratory Animals of the National Institutes of Health. The protocol was approved by the Institutional Animal Care and Use Committee of the Ben-Gurion University of the Negev, Beer Sheva, Israel (Permit number: IL-58-09-2012); Mice were housed in sterile conditions under 12-h light/dark cycles and fed Purina Chow and tap water *ad libitum*.

Mice were immunized as described below and then intranasally inoculated with BSA under deep anesthesia using Isoflurane (Piramal Critical Care Inc., PA, USA). Mice were humanely sacrificed by CO_2_ asphyxiation as recommended by the AVMA guidelines for euthanasia 2013. The lungs were excised. In all experiments mice were humanely euthanized by CO_2_ asphyxiation if they become moribund or show evidence of distress. The following criteria were considered sufficient evidence of distress to warrant such intervention in order to minimize pain and suffering to animals: severe weight loss (20% body weight); reluctance or inability to move freely; appearance of bristle fur; social disengagement; refusal or inability to eat or drink. No analgesic treatment was provided as such treatment may alter the immune response and may independently affect the outcome of the experiments [30]. None of the animals required the use of human end point and none were monitored for distress.

### Allergen sensitization and challenge

Experimental asthma was induced by sensitizing the mice to chicken egg albumin (OVA) by intraperitoneal injection with 0.1 μg OVA (grade III, Sigma-Aldrich, St. Louis, Missouri, USA) and 1 mg aluminum hydroxide (alum) as an adjuvant twice, followed by two 25 μg OVA or saline intranasal challenges 3 days apart, starting 10 days after the second sensitization. In all experiments, mice were anesthetized 24 h after the final intranasal challenge and serum was taken. Subsequently, mice were killed and bronchoalveolar lavage was performed; the lungs were excised for histological and FACS measurements.

### Bronchoalveolar lavage

For bronchoalveolar lavage (BAL) the trachea was exposed to allow insertion of a catheter, through which the lung was filled and washed two times with 2.5 ml of PBS with 2.5% FCS. BAL samples were stored on ice. The samples were centrifuged for 10 min at 1200 rpm and the supernatant was transferred to tubes and stored at −70°C. 200 μl (Adjuvant samples) or 400 μl (OVA samples) of PBS were added to the cell pellet and only 100 μl was used in the cytospin centrifuge. Fixation of cells on slides was performed using 100% methanol. Thereafter cells were stained using Diff-Quik staining with Hema-Diff kit (Statlab, McKinney, TX). The lungs employed for BAL were not used for any other lung tissue analysis.

### Lung tissue

To obtain single-cell suspensions, lungs were dissected and incubated in 2 ml RPMI with 2.5% FCS (Gibco), containing 1 mg/ml collagenase type IV and DNase type IV. Incubation was for 1 h in 37°c with a magnetic stirrer. Cells were then transferred through a 70 μm cell strainer. Red blood cells were lysed using 1ml of red blood cell lysis buffer (Sigma, St. Louis, USA). Cells were then washed with 10 ml cRPMI and centrifuged for 10 min at 1200 rpm. Cells were counted using Adam cell counter.

### Chemokines assay

Chemokine protein levels in the BAL supernatants were measured by single cytokine ELISA DuoSet kits from R&D systems (CCL17 and CCL24).

### IgE in serum

OVA immunized mice and adjuvant immunized mice were bled from the eye on day 28 following immunization. The blood samples were collected in yellow serum tubes, kept in room temperature for 20 min and then centrifuged for 25 min at 13,000 rpm in 4°C. Serum was transferred to new tubes and stored at −70°C. The OptEIA Set Mouse IgE (BD Biosciences) was used to quantify the amount of total serum IgE.

### Histology and PAS staining

Lung samples were fixed in 4% paraformaldehyde, dehydrated in alcohol, cleared in xylene and embedded in paraffin. No perfusion fixation was performed prior to the lung histological analysis. Four-micron sections were stained with H&E using established protocols. Periodic acid-Schiff stain (PAS) staining was assessed using Bio-Opticakit (cat num. 04–130802). The lung sections were sent to a pathologist for quality analysis.

### Antibodies and Flow cytometry

Flow cytometry analysis was employed for analysis of cell surface marker expression. Lung cells, or bronchoalveolar lavage fluid (BALF) cells (0.5*10^6^ per well) were plated in 96-well U-bottom plates. Cells were washed and nonspecific binding was blocked with anti-CD16/CD32 in 0.5% FCS/0.5% mouse serum/PBS for 15 min on ice. Cells were then stained for 25 min with the following specific mAbs: Biotin-conjugated-anti-mF4/80, FITC-anti-mCD45, PE-anti-mNK1.1, pacific blue-anti-mCD11c, PE Cy7-anti-GR1, APC-anti-NK1.1, APC-anti human Fc-IgG, APC-conjugated streptavidin, all purchased from eBioscience. For staining with fusion mNCR1-Ig (Ly94-Ig), cells were incubated with 2–4 mg of the mNCR1-Ig for 2 h at 4°C then washed, and stained with APC-conjugated-F(ab’)2 goat-anti-human-IgG-Fc (109-136-098, minimal cross-reaction to bovine, horse and mouse serum proteins, Jackson Immuno Research, West Grove, PA). Staining and washing buffer consisted of 0.5% (w/v) BSA and 0.05% sodium azide in PBS. Propidium iodide or AmCyan dye was added prior to reading for exclusion of dead cells. Stained cells were analyzed using either FACS Calibur or FACS Canto II (Becton Dickinson, Mountain View). The data was then analyzed either with BD CELLQuest TM 3.3 software or FlowJo software version 6.3.4 (Tree Star). Fluorescence data was acquired using logarithmic amplification and reported fluorescence intensity units represent conversion of channel values according to the logarithmic scale (range 10^0^ to 10^4^). Results are shown as the geometric mean fluorescence intensity (MFI) of the stained populations.

### RT-PCR

Total RNA was extracted from mice lungs by RNA purification kit (RNeasy; Qiagen, Valencia, CA). Purified RNA was converted to cDNA using Maxima H Minus First Strand cDNA synthesis Kit (Thermo scientific, Waltham, MA, USA). Twenty 100 ng RNA was subsequently used as a template for each Real-Time-PCR reaction. Primers appear in S1 Table. All PCR reactions for cytokine-specific mRNA were performed using the ABI Prism 7500 sequence detection system (Applied Biosystems, Foster City, CA, USA). HPRT mRNA level served as an endogenous control. In initial studies, 5-fold dilutions of cDNA generated a linear signal curve over at least a 30-fold range of cDNA concentrations. mRNA induction in pulsed lung tissue was reported as fold increases over uninfected lung mice. PCR results were analyzed with SDS 2.02 software (Applied Biosystems).

### Statistical analysis

Results obtained from groups of 3 to 12 mice were expressed as the mean±SD. Differences in cell numbers between two groups were analyzed using two-tailed Student’s *t*-test or by one-way ANOVA. *p* values less than *p* < .05 were considered to be statistically significant.

## Results

### NCR1 involvement in IgE production in OVA/alum immunized mice

**Fig 1:**
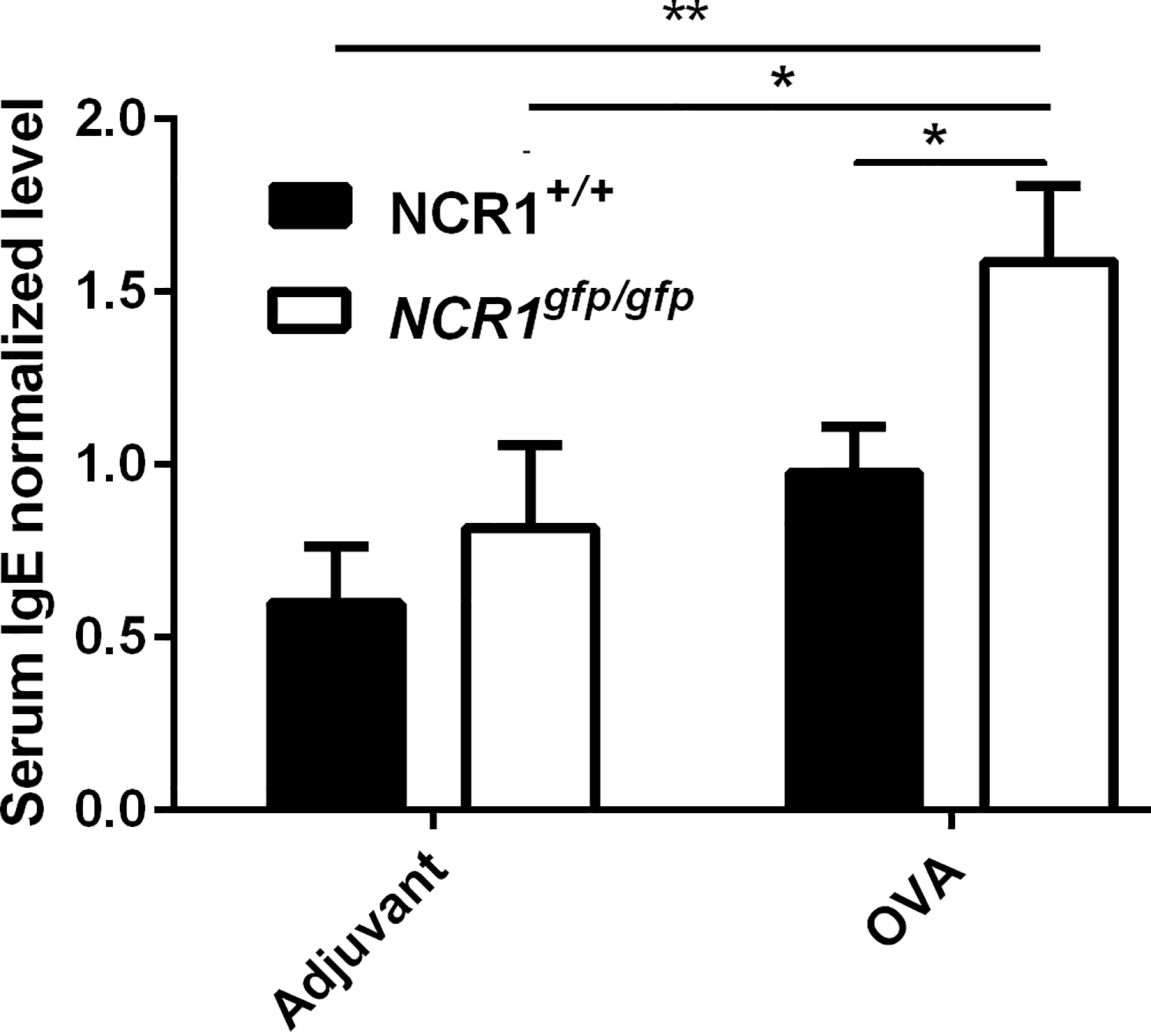
Increased IgE production in the absence of NCR1. *NCR*1^*+/+*^ and *NCR1*^*gfp/gfp*^ C57Bl/6 mice were i.p. immunized with either OVA/Alum or adjuvant only on days 0 and 14. Ten days after the second immunization, mice were challenged twice intranasally with OVA at days 24 and 27. Twenty four h following the second challenge, serum was drawn for measurement of the total IgE levels (n = 8 to 11). Results are the summary of 2 independent experiments and were normalized according to the OVA immunized *NCR1*^*+/+*^ group average that was considered as 1 in each experiment. *p < 0.05; **p < 0.01 1 way ANOVA multiple comparisons, GraphPad)

Allergic airway inflammation is characterized by the induction of IgE in the serum. OVA/Alum immunization is a model of allergic airway inflammation in mice. A normalized summary of two experiments and representative experiment are presented to strengthen the results. In the normalized summary of the two experiments, *NCR1*^*gfp/gfp*^ mice demonstrated increased levels of total serum IgE following OVA immunization compared to adjuvant immunized *NCR1*^+/+^ and *NCR1*^*gfp/gfp*^ mice (Fig 1; *p*<0.01; *p*<0.05, respectively. OVA/alum immunized *NCR1*^+/+^ mice IgE mean levels were considered as 1). OVA immunized *NCR1*^*gfp/gfp*^ mice demonstrated an increase in IgE serum levels in comparison to the adjuvant immunized *NCR1*^*gfp/gfp*^ mice (Fig 1 p < 0.05). An increase in IgE levels were also observed in *NCR1*^+/+^ mice, however this trend did not reach significance (p < 0.88). Most importantly, *NCR1*^*gfp/gfp*^ mice demonstrated significantly increased levels of total serum IgE following OVA immunization compared to OVA immunized *NCR1*^+/+^ mice (Fig 1 p < 0.05). Thus, NCR1 expression reduces allergic inflammation response.

In the representative experiment adjuvant immunized *NCR*1^*+/+*^ mice demonstrated significantly higher IgE levels than those observed in *NCR1*^*gfp/gfp*^ mice (p < 0.01). Following OVA immunization the levels of IgE increased significantly in the *NCR1*^*gfp/gfp*^ mice in comparison to adjuvant immunized mice (p < 0.05). In addition, following OVA immunization the levels of IgE were significantly higher in *NCR1*^*gfp/gfp*^ in comparison to *NCR*1^*+/+*^ mice (p < 0.01). These results suggest that NCR1 is involved in preventing excess IgE expression.

### NCR1 involvement in inflammation in OVA/alum -immunized mice

**Fig 2:**
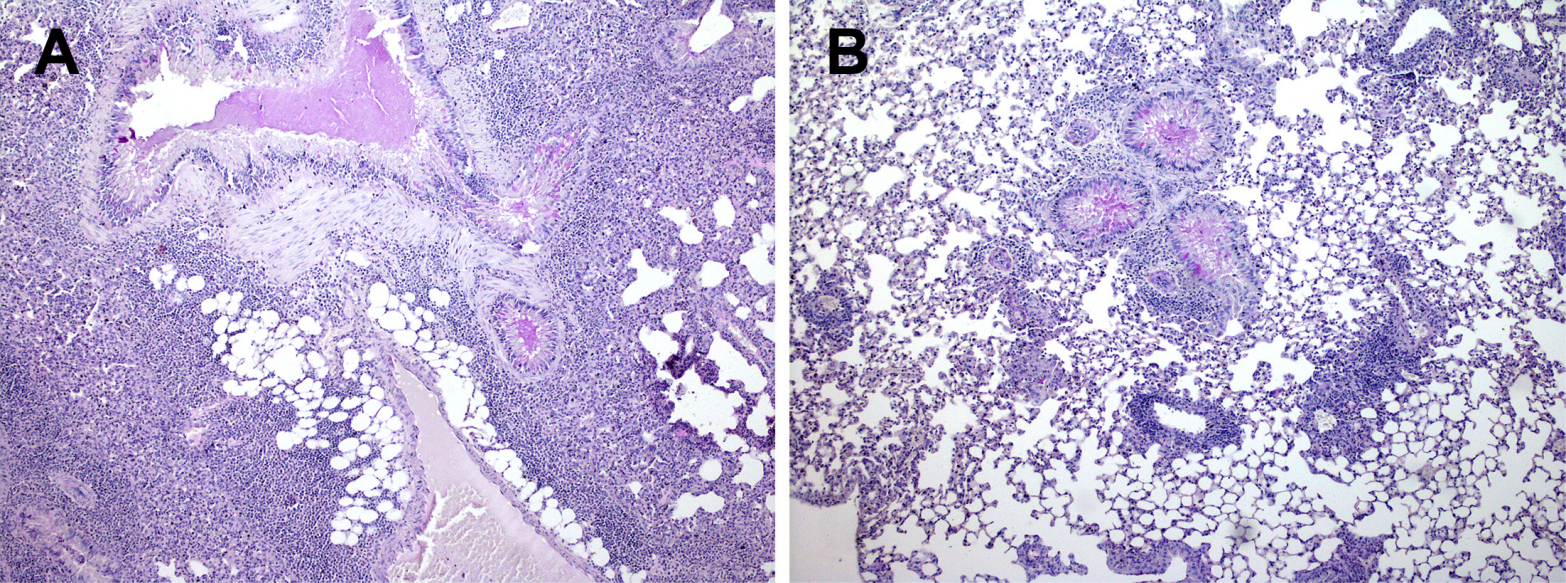
Increased inflammation in the lungs of *NCR1*^*gfp/gfp*^ mice H&E+PAS x180). *NCR1*^*gfp/gfp*^ C57Bl/6 mice were intraperitoneally (i.p) immunized with either OVA/alum or adjuvant only on days 0 and 14. Ten days after the second immunization mice were challenged twice intranasally with OVA at days 24 and 27. Twenty-four hours following the second immunization mice were euthanized and the left lungs were harvested for histology and RT-PCR. Representative Hematoxylin& Eosin (H&E) and PAS stained sections from each group are shown. Inflammation severity was assessed blindly by the pathologist. (A) OVA/alum immunized *NCR*^*gfp/gfp*^ (H&E+PAS x180). (B) Alum immunized *NCR*^*gfp/gfp*^ (H&E+PAS x180).

**Fig 3:**
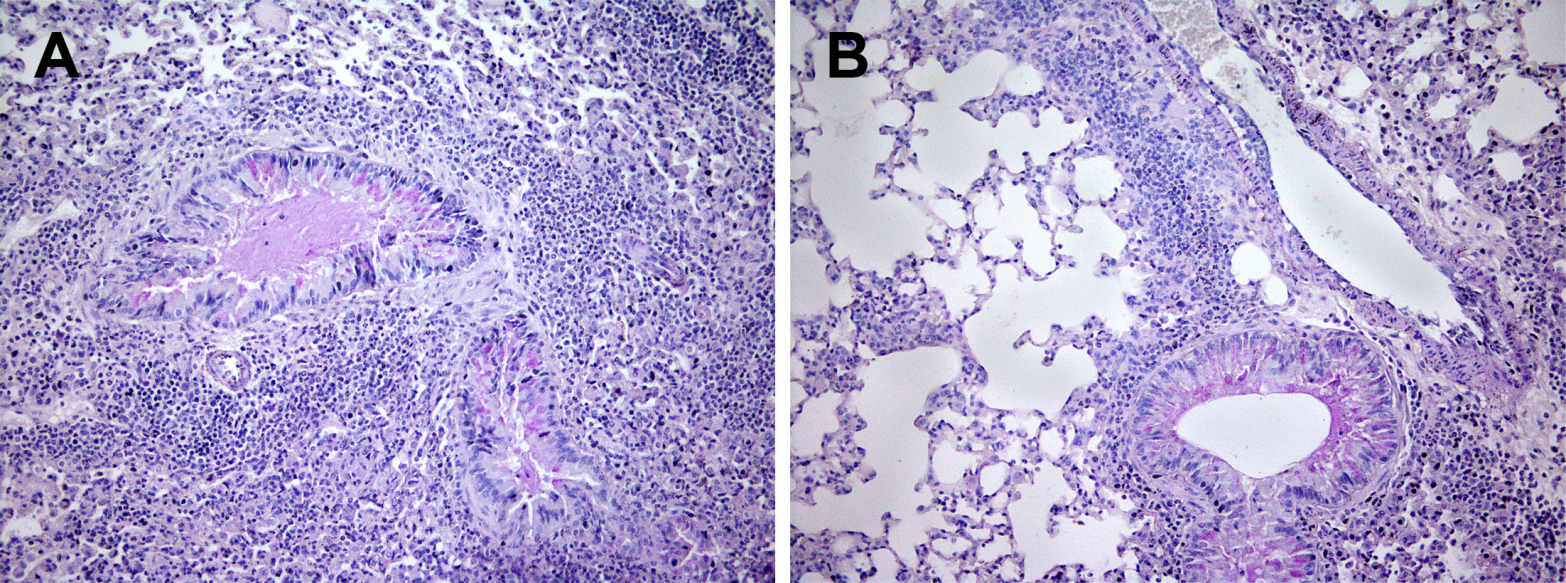
Increased inflammation in the lungs of *NCR1*^*gfp/gfp*^ mice (H&E+PAS x270). Mice were immunized and samples were process as described in Fig 2. (A) OVA immunized *NCR*^*gfp/gfp*^ (H&E+PAS x270). (B) Alum immunized *NCR*^*gfp/gfp*^ (H&E+PAS x270).

**Fig 4:**
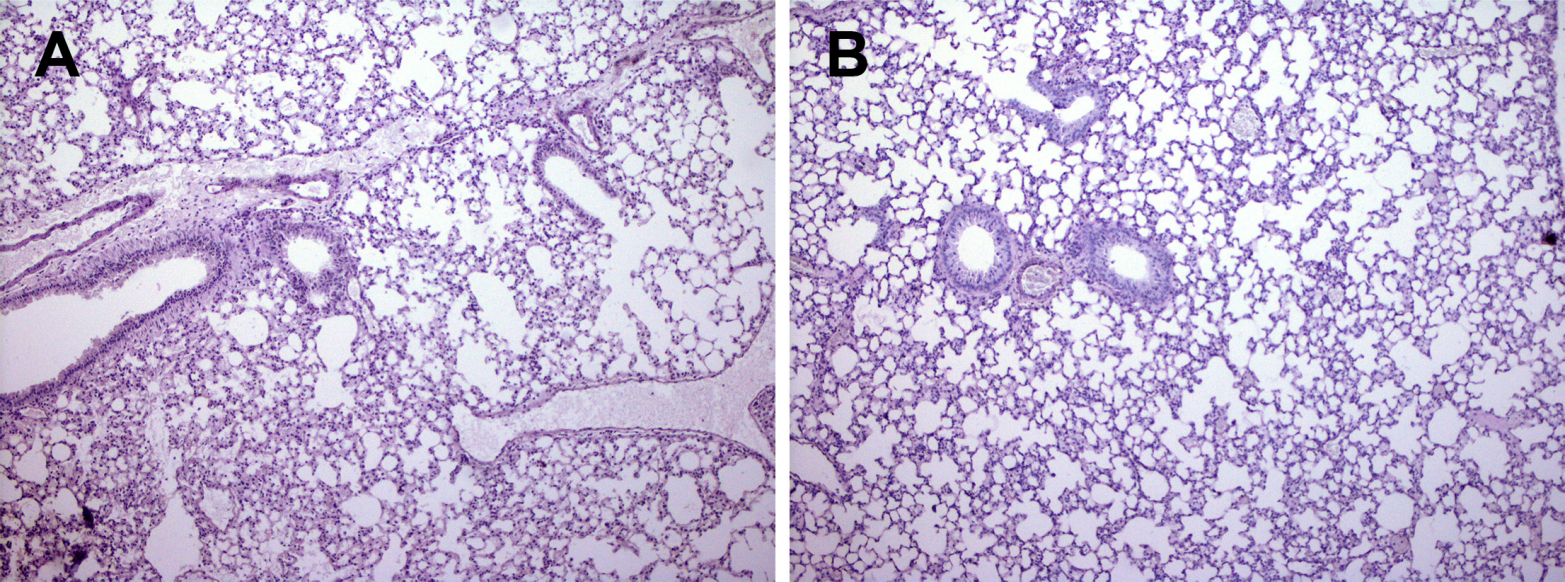
Increased inflammation in the lungs of *NCR1*^*+/+*^ mice (H&E+PAS x180). *NCR1*^*+/+*^ mice were immunized and samples were process as describe in Fig 2. (A) OVA/Alum immunized *NCR*^*+/+*^ (H&E+PAS x180). (B) Alum immunized *NCR*^*+/+*^ (H&E+PAS x270).

**Fig 5:**
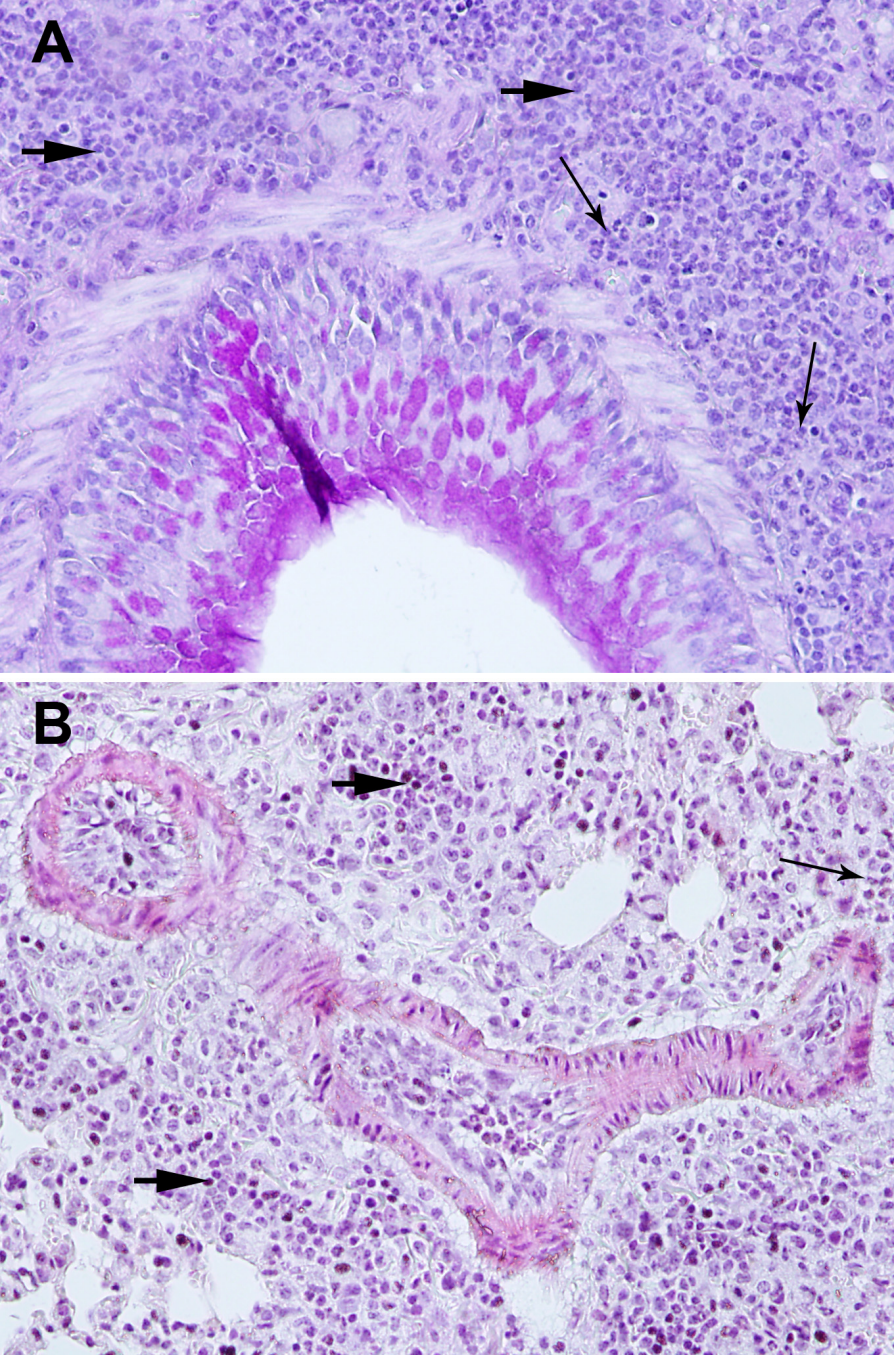
Increased inflammation in the lungs of *NCR1*^*gfp/gfp*^ mice compared to *NCR1*^*+/+*^ mice (H&E+PAS x340). Mice were immunized and samples were process as described in Fig 2. (A) OVA immunized *NCR*^*gfp/gfp*^ (H&E+PAS x340). (B) OVA/Alum immunized *NCR*^*+/+*^ (H&E+PAS x340). A thick arrow with a short tail identifies mononuclear cells and thin arrow with a long tail identifies polymorphonuclear cells.

To understand the nature of inflammation, we preformed lung H&E histology together with Periodic acid-Schiff stain (PAS) for assessment of lung structure, extent of immune cell infiltration and mucus secretion from goblet cells. The lung structure of OVA/Alum immunized *NCR1*^*gfp/gfp*^ mice was not preserved (Fig 2A; x180, Fig 3A; x270) in contrast to the lung structure of OVA/alum immunized *NCR1*^+/+^ mice (Fig 4A; x180,). *NCR1*^+/+^ mice lung structure was preserved similarly to the lung structure of adjuvant immunized *NCR1*^*gfp/gfp*^ and *NCR1*^+/+^ mice (Fig 2B and Fig 4B, respectively; x180). An overwhelming peribronchial and perivascular infiltration composed of granulocytes and mononuclear cells (lymphocytes and macrophages) was observed in the OVA/alum immunized *NCR1*^*gfp/*gfp^ mice (Fig 2A and Fig 3A). Peribronchial and perivascular infiltration of immune cells was also observed in the OVA/alum immunized *NCR*^*+/+*^ mice, albeit to a much lower extent than in the OVA immunized *NCR1*^*gfp/gfp*^ mice (Figs 4A and 2A. respectively). No infiltration of immune cells could be found in the adjuvant immunized *NCR1*^*gfp/gfp*^ and *NCR*^*+/+*^mice (Figs 2B and 4B, respectively). PAS staining levels were similar in OVA *NCR1*^*gfp/gfp*^ and *NCR*^*+/+*^ mice (Fig 2A and Fig 4A, respectively) and in both it was higher than that observed in adjuvant immunized mice. A better identification of the polymorphonuclear and mononuclear cells was obtained at a higher magnification (x340) of the histological slides of the lungs obtained from *NCR1*^*gfp/gfp*^ and *NCR*^*+/+*^ mice (Fig 5A and 5B, respectively). Allergen-challenged mice demonstrated significant increased expression of the mucin genes MUC5A/C and Gob5 in comparison to adjuvant immunized mice (S2A Fig, *p* < .05; S2B Fig *p* < .001, respectively) with no significant difference between *NCR1*^*+/+*^ and *NCR1*^*gfp/gfp*^ mice, which confirm that mucin gene expression is NCR1 independent.

### NCR1 involvement in eosinophil and macrophage infiltration to the lung

**Fig 6:**
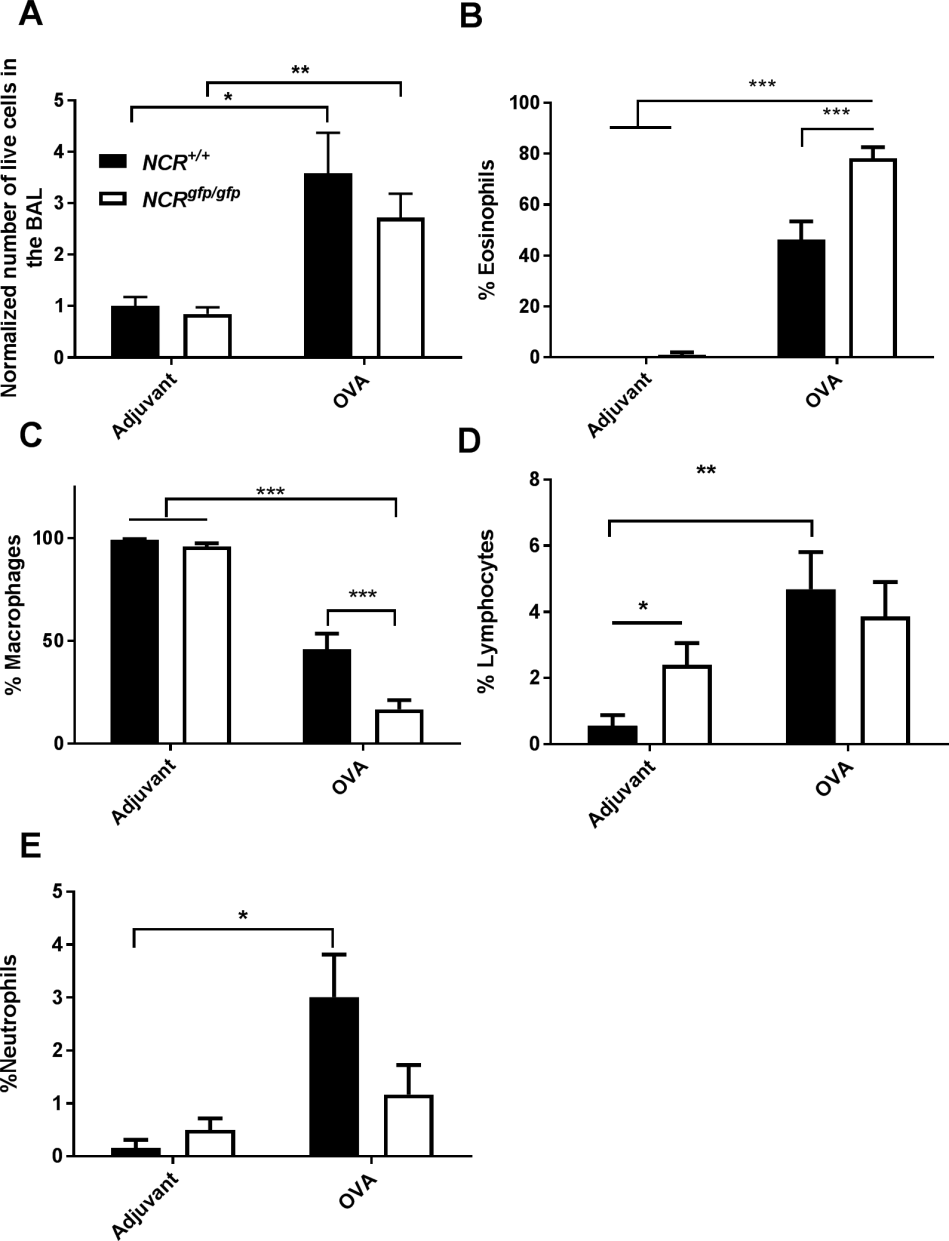
NCR1 is involved in eosinophil and macrophage infiltration to the lung. *NCR1*^*+/+*^ (Adjuvant n = 8, OVA n = 10) and *NCR1*^*gfp/gfp*^ (adjuvant n = 10, OVA n = 11) mice were immunized with either OVA or adjuvant as described in the Materials and Methods section. (A) The BAL was lavaged from each mouse, stained with PI and analyzed by flow cytometry for live cell count. The bar graph represents normalized number to the adjuvant immunized *NCR1*^*+/+*^ group whose average was considered as 1 of live cells in the BAL. (B-E) The bar graph represents an average percentage of eosinophils (B), macrophages (C), lymphocytes (D), and neutrophils (E) in the lung BAL (percentage of cells ± SEM; two-tailed Student *t*-test). These are the combined results of 2 experiments performed at different time points. * p < 0.05, **p < 0.01 *** p < 0.001.

We further explored whether NCR1 affects the composition of cells in the lung. Following OVA immunization, the total number of immune cells in the lung BAL increased considerably for both *NCR1*^+/+^ and *NCR1*^*gf*p/gfp^ mice in comparison to adjuvant immunized mice (Fig 6A; p<0.05 and p<0.01, respectively). The results are presented in Fig 6. Results from two experiments were normalized and summed to obtain better statistical power (the mean number of cells of adjuvant immunize *NCR1*^+/+^ mice was considered as 1). The increased cell number in OVA immunized *NCR*^*+/+*^ and *NCR1*^*gfp/gfp*^ mice in the lung BAL is characterized by a higher percentage of eosinophils in comparison to their respective adjuvant immunized control mice (Fig 6B; p < 0.001). OVA-immunized *NCR*^*gfp/gfp*^ mice demonstrated a significant increase in the percentage of eosinophils in comparison to OVA-immunized *NCR1*^+/+^ mice (Fig 6B; p < 0.01).

No differences in the percentage of macrophage were observed between the adjuvant immunized *NCR*^*+/+*^ and *NCR1*^*gfp/gfp*^ mice. However, a decrease in the percentage of macrophages was observed in OVA immunized *NCR*^*+/+*^ and *NCR1*^*gfp/gfp*^ mice in comparison to adjuvant-immunized *NCR*^*+/+*^ and *NCR1*^*gfp/gfp*^ mice (Fig 6C; p < 0.001). Moreover, the decrease in number of the macrophages in the lung of *NCR1*^*gfp/gfp*^ was significantly higher than in the OVA immunized *NCR*^*+/+*^ mice (Fig 6C p < 0.001). Adjuvant immunized *NCR1*^*gfp/gf*p^ mice demonstrated an significant increased percentage of lymphocytes compared to adjuvant immunized *NCR1*^+/+^ mice (Fig 6D p < 0.01). OVA-immunized *NCR*^*+/+*^ mice demonstrated a significant increase in the percentages of lymphocytes in comparison to adjuvant immunized *NCR*1^*+/+*^ mice and *NCR1*^*gfp/gfp*^ mice (Fig 6D; p < 0.05). The increase in the percentage of lymphocytes in the OVA-immunized *NCR1*^*gfp/gfp*^ mice did not reach significance in comparison to the adjuvant immunized *NCR1*^*gfp/gfp*^ mice. In contrast to the variation in eosinophils and macrophages, the percentage of lymphocytes did not differ significantly between allergen-challenged *NCR*1^*+/+*^ and *NCR1*^*gfp/g*fp^ mice. The increase in the percentage of neutrophils observed in OVA-Immunized *NCR1*^*+/+*^ and *NCR1*^*gfp/gfp*^ mice did not reach significance in comparison to the adjuvant immunized controls. Together, these results indicate that NCR1 expression is involved in decreased allergic inflammation namely decreased percentage of eosinophils in the BALF. In the absence of NCR1, allergic inflammation results in increased percentage of eosinophils, which is accompanied with a decreased percentage of macrophages.

Results from a representative experiment in which the actual cell number in the lung are presented in S3 Fig. The results are similar to those presented in Fig 6 concerning the numbers of eosinophils and macrophages in the BAL. In S3 Fig we added a panel demonstrating the percentages of dead cells in the BAL. No increase in the percent of dead cells could be observed following OVA immunization in comparison to adjuvant immunization in both *NCR1*^*+/+*^ and *NCR1*^*gfp/gfp*^.

### NCR1 and its ligand expression during allergic airway inflammation

To determine NCR1 and its ligand expression in the lungs, The following flow cytometric strategy was used to define different subpopulations of cells in the lung parenchyma. We first determined the number of total live immune cells in the lung tissue (parenchyma) using anti CD45 antibodies and PI by flow cytometry. Single cells were gated to exclude doublets by size. CD45 positive and 7AAD (a marker for dead cells) negative cells were then further gated on CD11c and GR1 markers. Five populations were gated according to these markers: 1. CD11c^+^GR1^-^ cells (myeloid DCs), 2. CD11c^++^GR1^-^ cells (lung DCs), 3. CD11c^+^GR1^+^ cells (plasmacytoid Dc), 4. CD11c^-^GR1^+^ cells, 5. CD11c^-^GR1^int^ cells. The populations were then gated on Sigelic F marker and SSC to distinguish between eosinophils and neutrophils. CD11c^-^GR1^+^ cells were divided into two sub-populations: 52% were sigelic F^-^SSC^low^ cells and 38% were sigelic F^-^ssc^high^ cells (neutrophils). CD11c^-^GR1^int^ cells were 27% Sigelic F^+^SSC^high^ cells (eosinophils). Ly94-Ig analysis was performed on each of the populations (S4 Fig 1–5).

**Fig 7:**
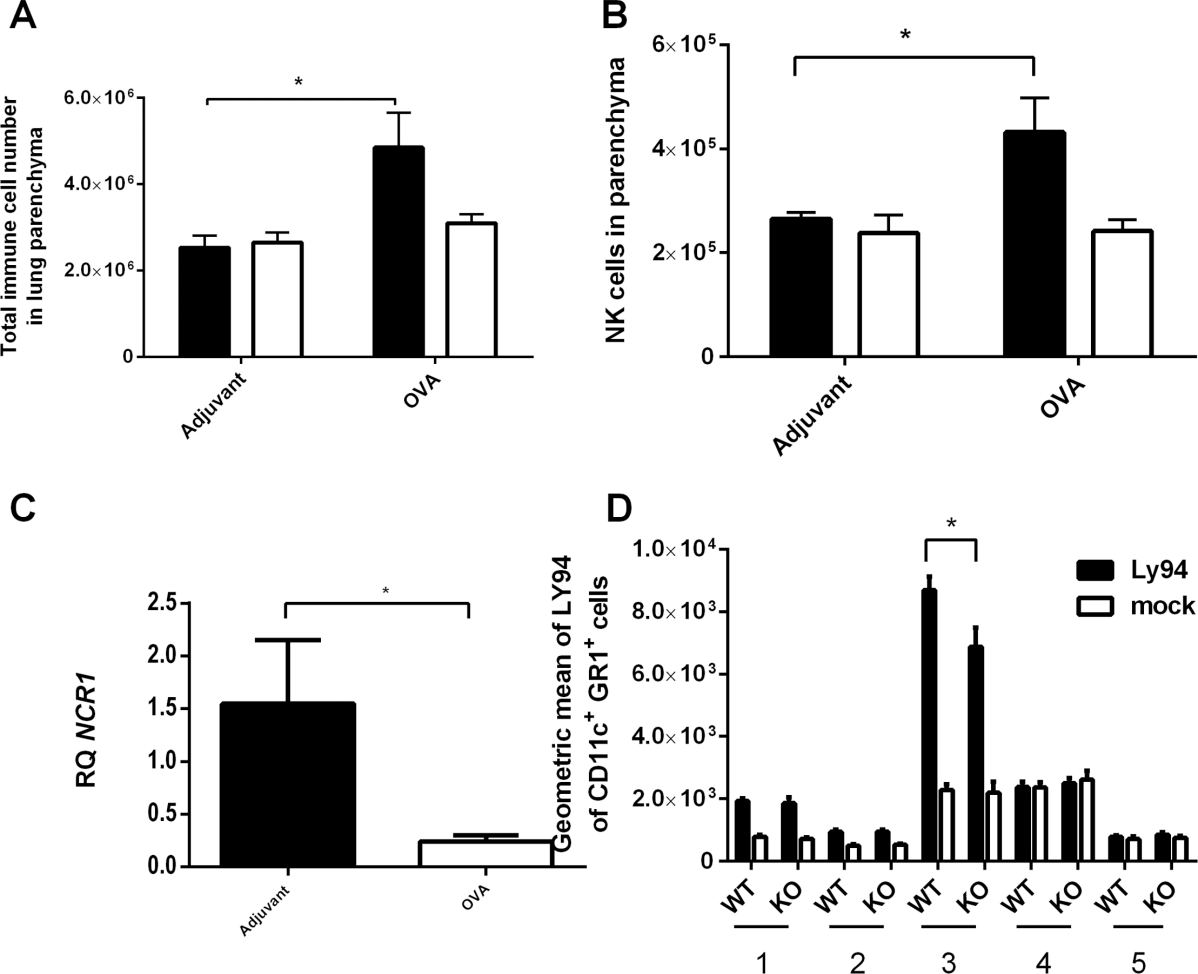
A decrease in NCR1 mRNA expression and increase of NCR1 ligand expression in the BAL of OVA immunized mice. *NCR*^*+/+*^ and *NCR*^*gfp/gfp*^ mice were immunized with either adjuvant alone or with OVA/alum as described in the Material and Methods section. The mice were euthanized and the lung tissue was dissociated into single cells. (A) Total number of live immune cell. (B) NK cell number as determined by anti NK1.1 antibody. (C) qRT PCR performed with *NCR1* appropriate primers (S1 Table). (D) Five populations of cells carrying or lacking CD11c or GR1 were determined and stained with LY94-Ig fusion protein. *p > 0.05.

Following OVA immunization, an increase in the total live immune cells was observed in the lung parenchyma (Fig 7A). To determine whether differences in the number of NK cells can be observed between the *NCR*^*+/+*^ and *NCR*^*gfp/gfp*^ mice, anti-NK1.1 antibody was used. No differences in the NK cell number could be observed between *NCR*^*+/+*^ and *NCR*^*gfp/gfp*^ mice following adjuvant immunization. However, following OVA immunization a significant increase in the NK cell number was observed in *NCR*^*+/+*^ mice, but no alteration in NK cell were observed in *NCR*^*gfp/gfp*^ mice (Fig 7B, p < 0.05). To determine the extent of *NCR1* expression in adjuvant or OVA immunized *NCR*^*+/+*^ mice, qRT PCR was used. While there were no differences in *NCR1* expression between the adjuvant immunized mice (data not shown), a significant decrease in *NCR1* expression was observed in OVA immunized *NCR*^*+/+*^ mice (Fig 7C, p < 0.05) in comparison to the adjuvant immunized mice.

Following OVA immunization, NCR1 ligand was significantly increased in the *NCR*^*+/+*^ mice in comparison to *NCR*^*gfp/gfp*^ mice in one population. The major population that demonstrates an increase in NCR1 ligand expression was CD11c^+^GR1^+^ expressing cells, which constitutes the plasmacytoid DCs in the lungs (Fig 7D).

### Increased allergy related chemokine production in the lungs of *NCR1*^*gfp/gfp*^ mice

**Fig 8:**
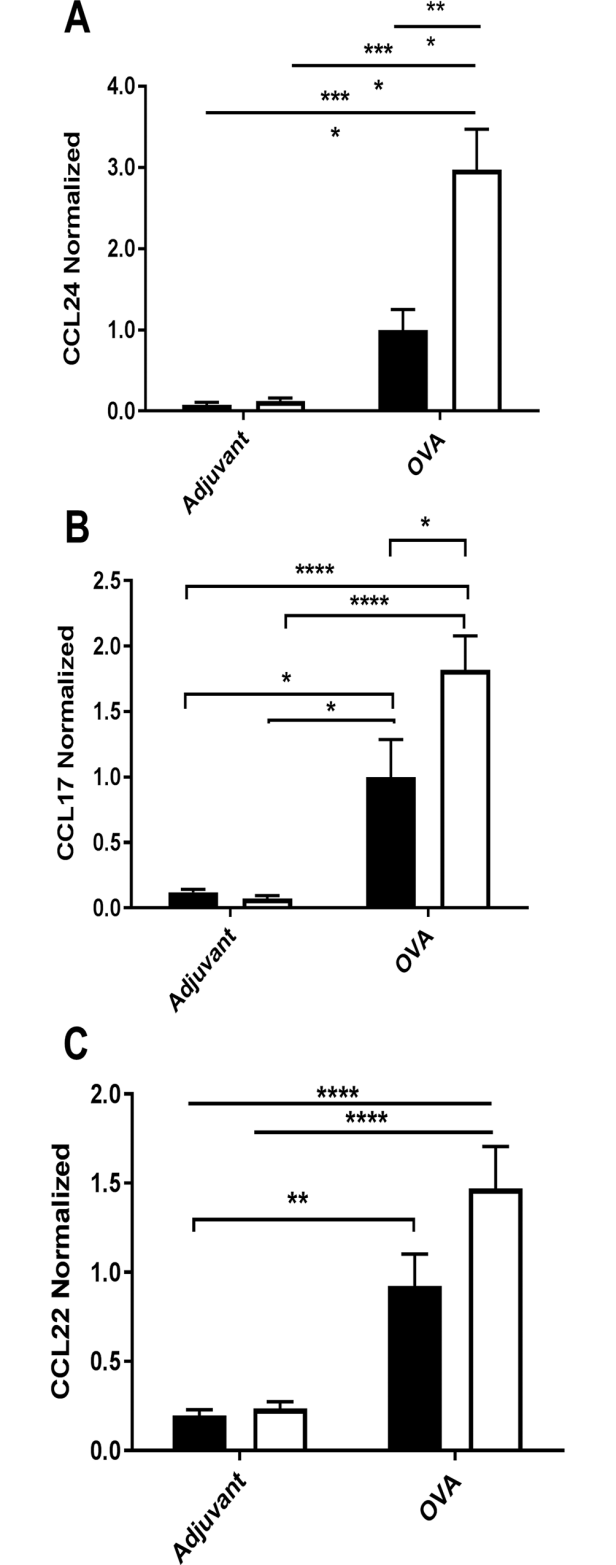
Increased allergic airway inflammation in the lungs of *NCR*^*gfp/gfp*^ mice. *Ncr1*^*+/+*^ and *Ncr1*^*gfp/gfp*^ *C57Bl/6* mice were i.p. immunized with either OVA or adjuvant on days 0 and 14. Ten days after the second immunization, mice were challenged twice intranasally with OVA at days 24 and 27. 24 h following the second challenge, BALF was taken from each mouse and used in ELISA to detect levels of (A) CCL24 (n = 8 to 11), (B) CCL17 (n = 9 to 10) and CCL22 (n = 9–10). Results are the summary of 3 independent experiments and were normalized according to the OVA *NCR1*^*+/+*^ ± SEM group average that was considered as 1 in each experiment. **p<0.01, ***p<0.001 (two-tailed Student *t-*test).

Allergic airway inflammation is characterized by the induction of chemokines, which are responsible for eosinophils (CCL24) and Th2 cells recruitment (CCL17 and CCL22). The protein concentration of these chemokines was determined in the BALF. In our model the level of CCL24, CCL17 and CCL22 chemokines found in the BALF of OVA immunized *NCR1*^*+/+*^ and *NCR*^*gfp/gfp*^ mice was increased significantly as compared to the levels demonstrated in adjuvant immunized *NCR1*^*+/+*^ and *NCR1*^*gfp/gfp*^ mice (CCL24; Fig 8A, *p*<0.01 and *p*<0.001, respectively and CCL17; Fig 8B, *p*<0.01 and *p*<0.001; Fig 8C, CCL22, p < 0.01 and p < 0.0001 respectively). Yet, consistent with increased peribronchial and perivascular infiltration of granulocyte and mononuclear cells (Fig 2, Fig 3, Fig 4, Fig 6 and S3 Fig), the expression of CCL24 and CCL17 was significantly higher in OVA immunized *NCR1*^*gfp/gfp*^ mice in comparison to OVA immunized *NCR1*^*+/+*^ mice (CCL24; Fig 8A, *p*<0.001; CCL17; 8.B, *p*>0.01, respectively). Together, these results demonstrated that in the presence of NCR1 a reduction in the exaggerated allergic inflammation is CCL24 and CCL17 dependent.

A representative experiment demonstrates the actual ELISA measurements (S5 Fig). Similarly to the combined experiment, an increase in the chemokine expression following OVA immunization could be observed in OVA immunized *Ncr1*^*+/+*^ and *Ncr1*^*gfp/gfp*^ (CCL24, S5A Fig p < 0.001 and p < 0.0001 respectively, CCL17, S5B Fig p<0.05 and p < 0.0001 and CCL22, S5C Fig p < 0.05 and p < 0.05). Significant increased chemokine production in *NCR*^*gfp/gfp*^ mice in comparison to *NCR*^*+/+*^ mice could be observed only for CCL24 and CCL17 (S5A Fig p < 0.01 and S5B Fig, p < 0.05, respectively).

### Increased Th2 type immune cytokines in response to allergic airway inflammation

**Fig 9:**
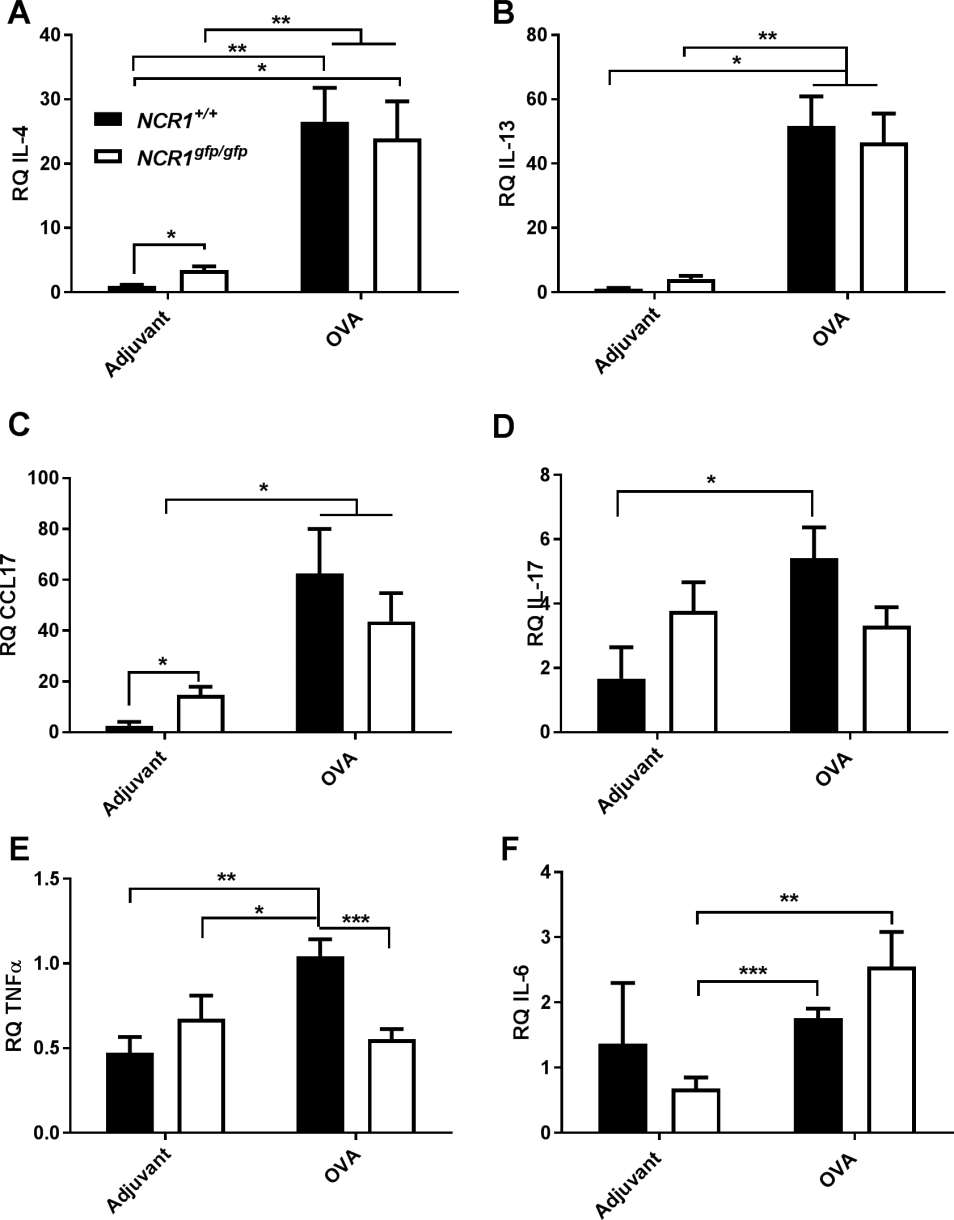
NCR1 involvement in chemokine and Th2, type immune cytokines in response to allergic airway inflammation. *NCR1*^*+/+*^ and *NCR1*^*gfp/gfp*^ mice were immunized with either OVA or adjuvant as described in Material and Methods, and 24 h following the last challenge the lungs were harvested and taken for RT-PCR. The bar graph represents levels of cytokine mRNA following either adjuvant or OVA immunization. Levels of cytokine mRNA were analyzed by RT-PCR and calibrated to mRNA level of HPRT (n = 3–7). (A) IL-4, (B) IL-13, (C) CCL17 (D) IL-17; (E). TNFα; (F) IL-6. *p < 0.05, **p < 0.01, ***p < 0.001 compared with the adjuvant immunized *NCR1*^*+/+*^ group ± SD (two-tailed Student *t*-test).

We explored the induction level of Th2, Th17, and Th1 type cytokines expression in lung parenchyma following OVA immunization. The adjuvant immunized *NCR1*^*gfp/gfp*^ mice demonstrated significant increase in the level of IL-4 (Fig 9A; *p*<0.05) in comparison to the level found in the adjuvant immunized *NCR1*^+/+^ mice. There was a significant increase in the expression level in both allergen-challenged-*NCR1*^+/+^ and *NCR1*^*gfp/gfp*^ mice of IL-4 (Fig 9A; *p*<0.01; fold increment of ~20) and IL-13 (Fig 9B; *p*<0.05, *p*<0.01, respectively; fold increment of ~50) in comparison to adjuvant immunized mice. In addition, following OVA immunization, an increase in CCL17 mRNA could be observed in both OVA immunized *NCR1*^+/+^ and *NCR1*^*gfp/gfp*^, (Fig 9C; *p*<0.05) in comparison to adjuvant immunized mice. The IL-17 expression levels increased significantly in OVA immunized *NCR1*^+/+^ mice and no increase was observed in OVA immunized *NCR1*^*gfp/gfp*^ mice as compared to their respective controls (Fig 9D, *p*<0.05). Following OVA immunization a significant increase in TNFα was observed in OVA immunized *NCR1*^*+/+*^ mice in comparison to adjuvant immunized *NCR1*^*+/+*^ and *NCR1*^*gfp/gfp*^ mice (Fig 9E; *p*<0.01 and *p*<0.05, respectively). No significant increase in TNFα expression level could be observed in OVA immunized in comparison to adjuvant immunized *NCR1*^*gfp/gfp*^ mice. The expression level of TNFα OVA immunized *NCR1*^*+/+*^ mice was significantly higher than in *NCR1*^*gfp/gfp*^ mice (Fig 9E, *p*<0.001). IL-6 levels increased significantly in OVA sensitized *NCR1*^*gfp/gfp*^ in comparison to adjuvant immunize *NCR1*^*gfp/gfp*^ mice (Fig 9F, p<0.01, *p*<0.05, respectively). No significant increase in IL-6 expression was found in OVA immunized *NCR1*^*+/+*^ in comparison to its respective control, (Fig 9F, *p*<0.001). The increment of increase following OVA immunization for IL-2 and IFNγ was less than 2 in both *NCR1*^*+/+*^ and *NCR1*^*gfp/gfp*^ (data not shown).

Taken together, these results demonstrate a total increase in Th2 cytokines (Il-4 and IL-13) and CCL17 chemokine mRNA expression independent of NCR1 receptor in response to OVA immunization. The increase in IL-17 and TNFα cytokines expression following immunization with OVA was NCR1 dependent. IL-6 increased only in absence of NCR1 in the mice following immunization with OVA suggesting either no involvement of NCR1 or NCR1 dampens IL-6 expression. Indeed, no alteration in Th1 cytokines (IL-2 and IFNγ) were observed upon allergic airway inflammation as expected.

## Discussion

The importance of NK cells in the development of allergic inflammation is starting to emerge [7, 12, 13]. However, two contradictory roles for NK cells in allergic inflammation have been suggested [31]. To further understand the role of NK cells in allergic inflammation we studied the role of NCR1 in a model of experimental asthma, classified as type 1 hypersensitivity reaction, in mice.

Our major finding is that NCR1 dampens the allergic reaction following OVA/alum immunization and challenge with OVA. The hallmark of allergic inflammation is total increase in IgE expression [32]. The highest concentration of total IgE in the serum was demonstrated in *NCR1*^*gfp/gfp*^ mice following OVA immunization in comparison to OVA immunized *NCR1*^*+/+*^ mice. Moreover, in *NCR1*^*gfp/gfp*^ mice the lung architecture was not preserved in contrast to OVA immunized *NCR1*^*+/+*^, the adjuvant immunized *NCR1*^*gfp/gfp*^ and *NCR1*^*+/+*^ mice. Infiltration of immune cells to the lungs was higher in the mice receiving OVA/Alum immunization compared with Alum immunization alone, demonstrating the ability of OVA to induce airway inflammation as previously described [33]. Infiltration of immune cells can be in response to Alum, as Alum induces excess Th2 type immune response even in the absence of IL-4Rα signaling [34]. However, the significant differences observed between the OVA immunized and the adjuvant immunized mice confirmed the development of allergic inflammation in the current model of allergic hypersensitivity reaction. The overwhelming increase in peribronchial and perivascular infiltration primarily of granulocytes, together with mononuclear cells, was observed in the absence of *NCR1* in the mice, while NCR1-expressing mice demonstrated peribronchial infiltration primarily of mononuclear cells and increased perivascular infiltration of granulocytes albeit to a much lower extent than in NCR1 deficient mice. In addition, a characteristic phenotype of asthma is an excessive production of mucus in the airways [26]. In OVA immunized mice an increase in PAS staining and MUC5A/C and Gob5 gene expression was observed, albeit without NCR1 involvement.

Airway eosinophilia is a key feature of allergic airway inflammation [33] and NK cells were suspected to be involved in the development of allergy-induced airway inflammation; specifically, they were critically important for the induction of allergic eosinophilic airway disease [15]. In the current study, we show that in the lung BALF a total increase in the number of cells was observed following OVA immunization in comparison to adjuvant immunized mice, which was NCR1 independent. However, there were significant differences in the composition of the cells in the lungs of *NCR1*^*gfp/gfp*^ mice and *NCR1*^*+/+*^ mice. There was a significant increase in eosinophils and decrease in macrophages in *NCR1*^*gfp/gfp*^ mice in comparison to *NCR1*^*+/+*^ mice, further confirming that NCR1 expression dampens the allergic inflammatory response. An increased number of lymphocytes was observed in the adjuvant immunized *NCR1*^*gfp/gfp*^ in comparison to *NCR1*^*+/+*^ mice. In OVA immunized *NCR1*^*+/+*^ mice, an increase in T lymphocytes was observed in comparison to adjuvant immunized *NCR1*^*+/+*^ and *NCR1*^*gfp/gfp*^ mice. No significant increase in the number of lymphocytes was observed following OVA immunization in *NCR1*^*gfp/gfp*^ mice.

The question to be answered is whether NCR1 regulates the development of allergic airway inflammation during the sensitization phase or at the effector phase. During the sensitization phase, APCs present allergens to T-cells in order to induce differentiation to allergen-specific Th2 cells. Th2 cells induce an immunoglobulin switch in B cells that leads to IgE production [35–37]. Thus, decreased levels of IgE in the presence of NCR1 indicate that NCR1 dampens the development of allergic eosinophilic airway inflammation, likely by regulating the sensitization phase. In contrast to the protective effect against lung allergic hypersensitivity demonstrated by NCR1 in the current study, the NKG2D cytotoxic receptor was shown to augment allergic hyersensitivity response to house dust mites (HDM) in a mouse model. Mice lacking the NKG2D receptor were resistant to a challenge with HDM in contrast to wild type mice. The resistant NKG2D lacking mice could be turned into susceptible mice when supplemented with wild NK cells producing granzyme B, but not with CD3^+^ T-cells [38].

The current study demonstrates that there was a significant increase in NK cell number in the lung parenchyma of the *NCR*^*+/+*^ mice in the allergic eosinophilic airway inflammation mouse model following OVA immunization. However, a decrease in *NCR1* mRNA expression was observed in the OVA immunized mice. In addition, an increase in the surface expression of NCR1 ligand was observed in the OVA immunized *NCR*^*+/+*^ and *NCR*^*gfp/gfp*^ mice on CD11c^+^GR1^+^ cells. CD11c^+^GR1^+^ cells are suspected to be plasmacytoidal dendritic cells. In general, the extent of activating receptor expression correlates with the extent of the NK cytolytic activity toward NK-susceptible target cells [39, 40]. Thus, NK cells with high NCR surface density display strong cytotoxicity, whereas, NK cells with low density of NCRs on their surface demonstrate poor cytolytic activity [41]. This generally accepted statement has been challenged in several studies. It has been shown that NCRs are weakly expressed on freshly isolated human blood derived NK cells, whereas NKp30 and NKp46 were more abundant than NKp44. These results are consistent with another study that shows NKp30 and NKp46 are expressed on non-activated and activated NK cells, whereas NKp44 is expressed preferentially after *in vitro* activation [42, 43]. Moreover, following Hepatitis E virus infection an increased expression of NKp44, NKp46 on NK/NKT-like cells were associated with decreased cytolytic activity [44]. It should be also mentioned that in addition to the cytolytic function of NCR1, this receptor is also important for the cross talk with antigen presenting cells in general and DCs in particular, and affect the cytokine milieu that is created during the development of inflammation, as previously described [45]. Thus, there is not always a correlation between NCRs expression and their functionality. Activation of NCRs is dependent on lack of MHC I expression. However the activation or the inhibition of NCR1 on NK cells depends also on the extent of its NCR ligands expression [46]. The ligand for NCR1 in allergy is unknown and further studies need to explore the interaction of NCR1 and its ligand, and how it affects the course of inflammation occurring during allergic airway inflammation.

Different lung DC subsets induce different immune responses, such as immunity versus tolerance, or Th1 versus Th2 immune responses. Lung myeloid DCs play a major role in inducing allergic airway inflammation in response to allergen challenge [47]. Under the condition of allergen challenge, CD11c^low^CD11b^high^ lung DC subset is rapidly expanded and more prone to induce robust Th2 response as compared to a Th1-prone response induced by CD11c^high^CD11b^low^ lung DCs. Our results demonstrate increased CD11c^+^GR1^+^LY94^+^ cells in the lungs of OVA immunized *NCR1*^*+/+*^ mice, in comparison to *NCR1*^*gfp/gfp*^, and are in accordance to previous studies demonstrating that murine plasmacytoid DCs (CD11c^int^GR1^+^) located in mouse lymph nodes produce type I interferon and demonstrate tolerogenic potential [48]. Lung plasmacytoid cells were shown to suppress T cell division and effector T cell generation induced by myeloid DCs, confirming the involvement of plasmacytoid DCs in regulating lung inflammation [49]. DCs are important cells, which can orchestrate the immune response at the sensitization phase in allergic airway inflammation. The induction of this DC-subset by NK cells can consequently affect the adaptive immune response developed. Investigating the different DC subtypes in the lung during allergic airway inflammation can be a key to understanding the mechanism for the NCR1 influence on the development of the allergic airway response. Currently, our results suggest that the cross talk between the plasmacytoidal and NK cells through NCR1 following OVA/alum immunization and challenge cause an increase in NK cell number in the lungs of NCR^*+/+*^ mice contributing to the ameliorated allergic hypersensitivity response. No increase in the numbers of NK cells could be observed in mice lacking NCR1 following OVA immunization. Previously, we have specifically shown that NCR1 is detrimental for the NK-DC cross talk in the presence of a pathogen, and cytokine production diminishes in the absence of NCR1 [45].

Several studies assigned a proinflammatory effector function to NK cells based on the depletion of NK cells in the OVA allergic inflammation mouse model. In the first study using C57BL/6 mice, depletion of NK and NKT cells before immunization, but not prior to challenge, demonstrated reduced inflammatory responses in mice. The proinflammatory activity was directly attributed to NK, but not to NKT cells, since using NKT cell deficient-mice demonstrated the same characteristics as the wild type mice. Moreover, in this study no effect of NK cells was observed when NK cells were depleted during the sensitization phase [15]. In a different study using BALB/cByj mice, NK cell depletion during immunization or sensitization phases reduced airway inflammation [14]. In the latter two studies, the two immunizations were followed by multiple OVA sensitization sessions.

Other studies supported the anti-inflammatory function of NK and the inflammation pro-resolving function of NK cells. It has been shown recently that human naïve or IL-12 activated NK cells are capable of triggering neutrophil apoptosis *in vitro*. This neutrophil apoptosis occurred upon NKp46 contact, in a Fas dependent manner and was accompanied by NK cell degranulation. [17]. NK cells were also shown to induce eosinophil activation and apoptosis through a contact-dependent process, but DNAM-1, LFA-1, CD54, NKp30, NKp46, NKG2D and TNFα independent process [18]. Moreover, macrophage phagocytosis of apoptotic cells has been shown to increase IL-10 production and decrease pro-inflammatory cytokine production. The increase in the apoptotic cell phagocytosis is probably controlled by the specialized pro-resolving lipid mediators (SPMs) including, among others, Lipoxin A_4_ (LXA_4_) and Resolvin E1 (RvE1) [50]. Haworth and colleagues were the first to demonstrate that resolvin E1 has a strong anti-inflammatory activity and it regulates IL-23, IFNγ, Lipoxin A_4_ and promotes resolution of allergic airway inflammation [51]. This study was followed by a second study in which they demonstrate that eosinophils and T-cells were cleared concomitantly with the appearance of NK cells in the lungs and in the mediastinal lymph nodes. The clearance of the eosinophils and T-cells was delayed in NK depleted mice. In addition, the NK cells expressed CMKLR1, a receptor for the pro-resolving mediator, resolvin 1 [14, 16, 51, 52]. Several recent studies have described unique populations of tissue-associated CD3^neg^ innate lymphoid cells (ILCs) expressing NCRs, which lack classical NK cell functions such as cytotoxicity and IFNγ production. ILCs were originally identified in the murine intestine as NKp46^pos^ cells, phenotypically similar to lymphoid tissue inducer (LTi) [53, 54].

Recently, NCR1 was shown to regulate the development of delayed type hypersensitivity in a mouse OVA model [55][30]. However, their results show that the NCR1 increase delayed type hypersensitivity responses in mice. A possible explanation for this discrepancy is the fact that the model in their study was focused on delayed type IV hypersensitivity allergic reaction, which takes 48 to 72 h to develop and is mediated by T cells. The model used in our study is a type 1 allergic reaction which is mediated by eosinophils, DCs and mast cells. In addition, our study is focused on the first 24 h following the second lung sensitization, while their study is focused on the response developed following the fifth lung sensitization. Therefore, the comparison between these two studies must be done with caution.

CCL24 chemoattractants secreted from lung epithelial cells or DC’s, direct eosinophils, while CCL17 and CCL22 direct Th2 CD4^+^ T-cells to the site of inflammation[5, 56–64]. CCL24, CCL17 and CCL22 were increased in OVA immunized mice in comparison to the adjuvant immunized mice as expected in the current model for allergy. However, CCL24, CCL17 chemokines were significantly increased in the BAL following OVA immunization of *NCR1*^*gfp/gfp*^ mice in comparison to *NCR1*^*+/+*^ mice, suggesting that the absence of NCR1 caused an increase in eosinophil and Th2 cells recruitment to the lungs. The increase in CCL24 level in the lung following OVA immunization correlates with the increased percentage of eosinophils observed in the absence of NCR1. However, the increase in CCL17 does not correlate with the percentages of lymphocytes in the lung of OVA immunized NCR1 non-expressing mice, which did not differ from that observed in OVA immunized NCR1 expressing mice. The observed increase in the CCL22 in OVA immunized *NCR1*^*gfp/gfp*^ mice did not reach significance in comparison to OVA immunized *NCR1*^*+/+*^ mice. Thus, there might be additional mediators that control the migration of lymphocytes to the site of inflammation following OVA immunization [65–68].

In order to better understand the mechanism of regulation, we screened Th1, Th2, Th17 cytokine mRNA and CCL17 chemokine in the mouse lungs following OVA immunization. The strong elevation of IL-4, IL-13 and CCL17 mRNA following OVA immunization demonstrates an intense allergic airway response, which was NCR1 independent. However, CCL17 protein expression in the lung BALF was significantly increased in the absence of NCR1, suggesting that an increase in CCL17 protein production occurs albeit similar mRNA expression in the OVA immunized *NCR1*^*gfp/gfp*^ and *NCR1*^*+/+*^ mice.

IL-6 increased only in absence of NCR1 in the mice following immunization with OVA suggesting either no involvement of NCR1 or NCR1 dampens IL-6 expression. Indeed, no alteration in Th1 cytokines (IL-2 and IFNγ) were observed upon allergic airway inflammation as expected.

NCR1 has no role in regulating Th1 cytokines as no increase in Th1 cytokines and chemokine expression levels following OVA immunization could be observed. Th1 cytokines were originally regarded as potentially protective against asthma, which was considered a purely Th2 response [69]. In a different study, which used an ear swelling mouse model of inflammation, deletion of Th2 cytokines (IL-4 and IL-5) as well as deletion of Th1 cytokine (IFNγ) lead to a decreased eosinophil recruitment and decreased pathogenesis of ear swelling [70]. In our study, the increased expression of IFNγ and IL-2 was negligible in agreement with the previous results.

NK cells were suggested as a target for asthma therapy. In several studies, NK cells were suspected to augment allergic inflammation. In other studies, NK cells were shown to enhance neutrophil and eosinophil apoptosis, which may lead to the resolution of the inflammation. In agreement with the latter studies in the current study, we demonstrate for the first time that NCR1 receptor dampens the allergic response in the OVA model of asthma *in vivo* in mice.

## Supporting Information

S1 Fig Increased IgE production in the absence of NCR1. *NCR*1^*+/+*^ and *NCR1*^*gfp/gfp*^ C57Bl/6 mice were i.p. immunized with either OVA/Alum or adjuvant only on days 0 and 14. Ten days after the second immunization, mice were challenged twice intranasally with OVA at days 24 and 27. Twenty four h following the second challenge, serum was drawn for measurement of the total IgE levels. A representative experiment is presented (n = 5 to 6 animal in each group). *p<0.05; **p<0.01 compared with the *NCR1*^*+/+*^ OVA-immunized group ±SEM (1 way ANOVA multiple comparison GraphPad).
(TIF)

S2 Fig NCR1 involvement in mucus genes expression. The bar graph shows the average RQ of MUC5A/C and Gob5 mRNA level for each group of mice. Each bar represents mean ±SD of n = 3 to 7. ** *p*<0.01, ***p<0.001 compared with the *NCR1*^*+/+*^ adjuvant treated group (two-tailed Student *t-*test). Results are summarized from two different independent experiments.
(TIF)

S3 Fig NCR1 is involved in eosinophil and macrophage infiltration to the lung: actual cell counts. *NCR1*^*+/+*^ (Adjuvant n = 4, OVA n = 5) and *NCR1*^*gfp/gfp*^ (adjuvant n = 4, OVA n = 5) mice were immunized with either OVA or adjuvant, as described in the Materials and Methods section. The BAL was lavaged from each mouse, stained with anti CD45 and PI, and analyzed by flow cytometry for live immune cell count (A). The number of eosinophils (B) macrophages (C) was determined by a differential staining. Percent dead cells in the BAL (D). These results are from a representative experiment. Statistical analysis: two-tailed Student t-test. *p < 0.05; ***p < 0.001 ****p < 0.0001
(TIF)

S4 Fig Identification of BAL cells subsets by Flow cytometry. Single cells were gated to exclude doublets by size. CD45 positive and 7AAD (a marker for dead cells) negative cells were then further gated on CD11c and GR1 markers. Five populations were gated according to these markers: 1. CD11c^+^GR1^-^ cells (myeloid DCs), 2. CD11c^++^GR1^-^ cells (lung DCs), 3. CD11c^+^GR1^+^ cells (plasmacytoid Dc), 4. CD11c^-^GR1^+^ cells, 5. CD11c^-^GR1^int^ cells. The populations were then gated on Sigelic F marker and SSC to distinguish between eosinophils and neutrophils. CD11c^-^GR1^+^ cells were divided into two sub-populations: 52% were sigelic F^-^SSC^low^ cells and 38% were sigelic F^-^ssc^high^ cells (neutrophils). CD11c^-^GR1^int^ cells were 27% Sigelic F^+^SSC^high^ cells (eosinophils). Ly94-Ig analysis was performed on each of the populations (1–5).
(TIF)

S5 Fig Increased allergic airway inflammation in the lungs of *NCR*^*gfp/gfp*^ mice: ELISA measurements of chemokines. *Ncr1*^*+/+*^ and *Ncr1*^*gfp/gfp*^ *C57Bl/6* mice were i.p. immunized with either OVA or adjuvant on days 0 and 14. Ten days after the second immunization, mice were challenged twice intranasally with OVA at days 24 and 27. 24 h following the second challenge, BALF was taken from each mouse and used in ELISA to detect levels of (A) CCL24 (n = 8 to 11) and (B) CCL17 (n = 9 to 10) and (C) CCL22 (n = 9–10). **p<0.01, ***p<0.001 (One tail ANOVA multiple comparison).
(TIF)

S1 Table Quantitative Real Time PCR primers. (DOCX)
