# Supplementary figures and images for: Natural Killer Receptor 1 Dampens the Development of Allergic Eosinophilic Airway Inflammation
Source: PLoS One. 2016 Aug 31;11(8):e0160779. doi: 10.1371/journal.pone.0160779 (PMC5007051; doi:10.1371/journal.pone.0160779)

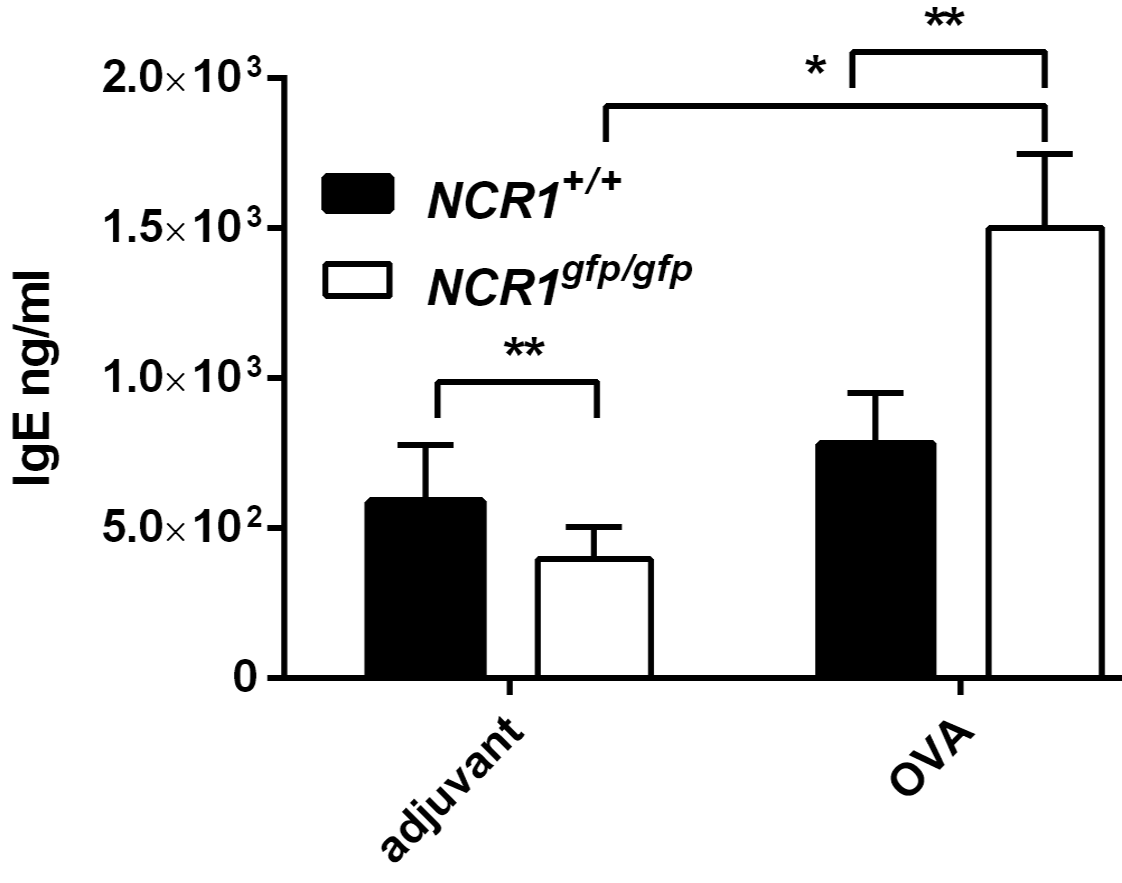

Supplement: S1 Fig — NCR1+/+ and NCR1gfp/gfp C57Bl/6 mice were i.p. immunized with either OVA/Alum or adjuvant only on days 0 and 14. Ten days after the second immunization, mice were challenged twice intranasally with OVA at days 24 and 27. Twenty four h following the second challenge, serum was drawn for measurement of the total IgE levels. A representative experiment is presented (n = 5 to 6 animal in each group). *p<0.05; **p<0.01 compared with the NCR1+/+ OVA-immunized group ±SEM (1 way ANOVA multiple comparison GraphPad). (TIF) [file pone.0160779.s001.tif]

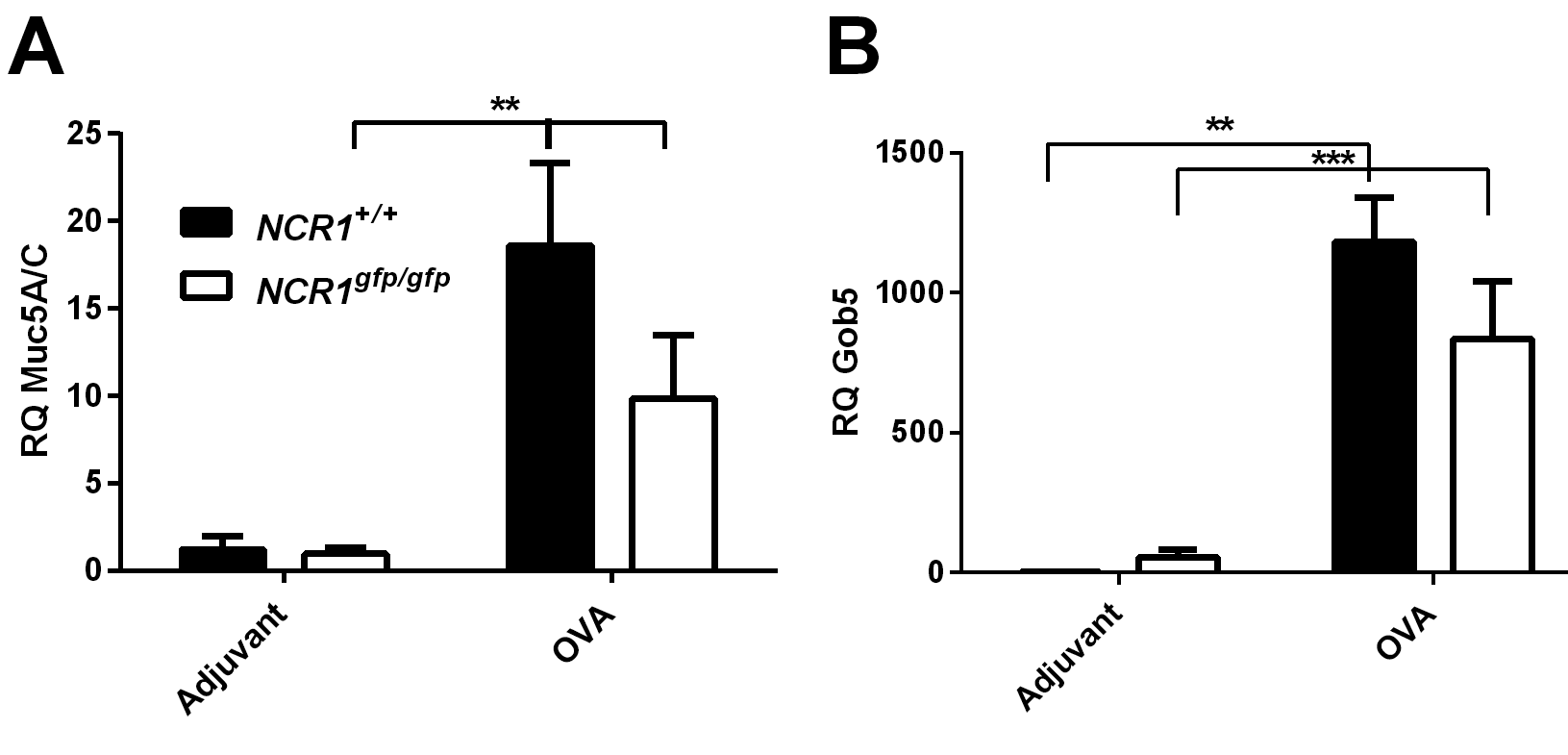

Supplement: S2 Fig — The bar graph shows the average RQ of MUC5A/C and Gob5 mRNA level for each group of mice. Each bar represents mean ±SD of n = 3 to 7. ** p<0.01, ***p<0.001 compared with the NCR1+/+ adjuvant treated group (two-tailed Student t-test). Results are summarized from two different independent experiments. (TIF) [file pone.0160779.s002.tif]

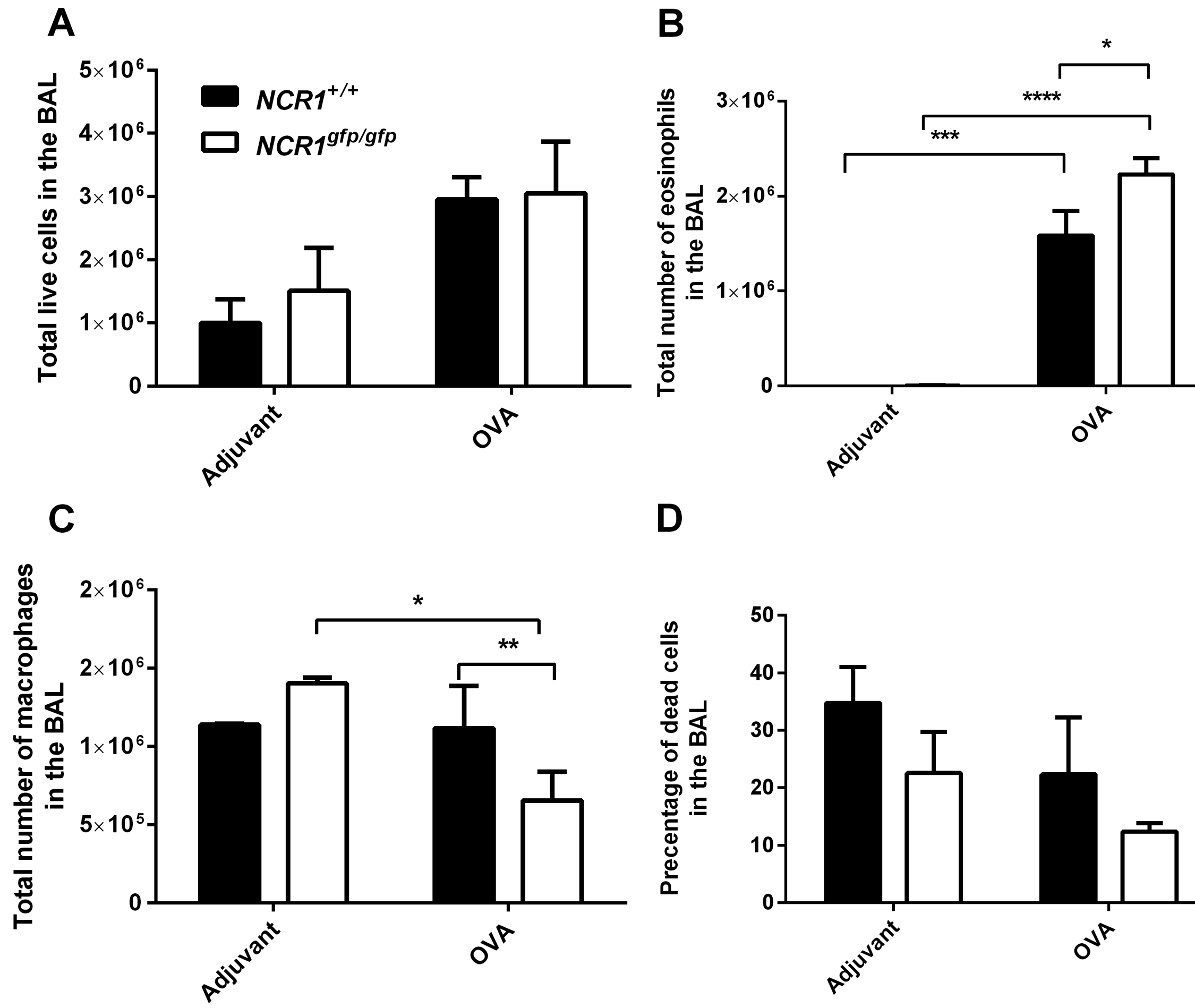

Supplement: S3 Fig — NCR1+/+ (Adjuvant n = 4, OVA n = 5) and NCR1gfp/gfp (adjuvant n = 4, OVA n = 5) mice were immunized with either OVA or adjuvant, as described in the Materials and Methods section. The BAL was lavaged from each mouse, stained with anti CD45 and PI, and analyzed by flow cytometry for live immune cell count (A). The number of eosinophils (B) macrophages (C) was determined by a differential staining. Percent dead cells in the BAL (D). These results are from a representative experiment. Statistical analysis: two-tailed Student t-test. *p < 0.05; ***p < 0.001 ****p < 0.0001 (TIF) [file pone.0160779.s003.tif]

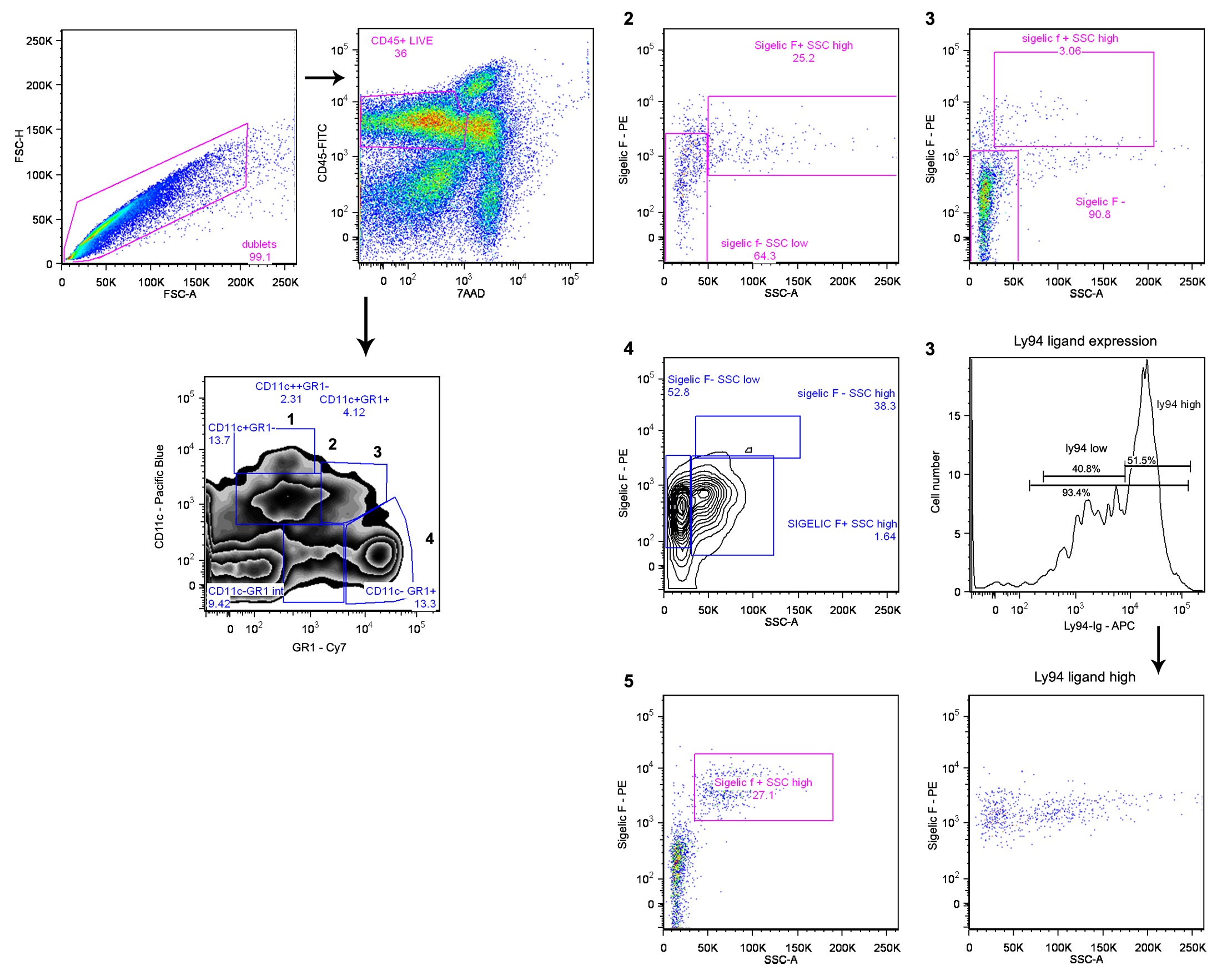

Supplement: S4 Fig — Single cells were gated to exclude doublets by size. CD45 positive and 7AAD (a marker for dead cells) negative cells were then further gated on CD11c and GR1 markers. Five populations were gated according to these markers: 1. CD11c+GR1- cells (myeloid DCs), 2. CD11c++GR1- cells (lung DCs), 3. CD11c+GR1+ cells (plasmacytoid Dc), 4. CD11c-GR1+ cells, 5. CD11c-GR1int cells. The populations were then gated on Sigelic F marker and SSC to distinguish between eosinophils and neutrophils. CD11c-GR1+ cells were divided into two sub-populations: 52% were sigelic F-SSClow cells and 38% were sigelic F-sschigh cells (neutrophils). CD11c-GR1int cells were 27% Sigelic F+SSChigh cells (eosinophils). Ly94-Ig analysis was performed on each of the populations (1–5). (TIF) [file pone.0160779.s004.tif]

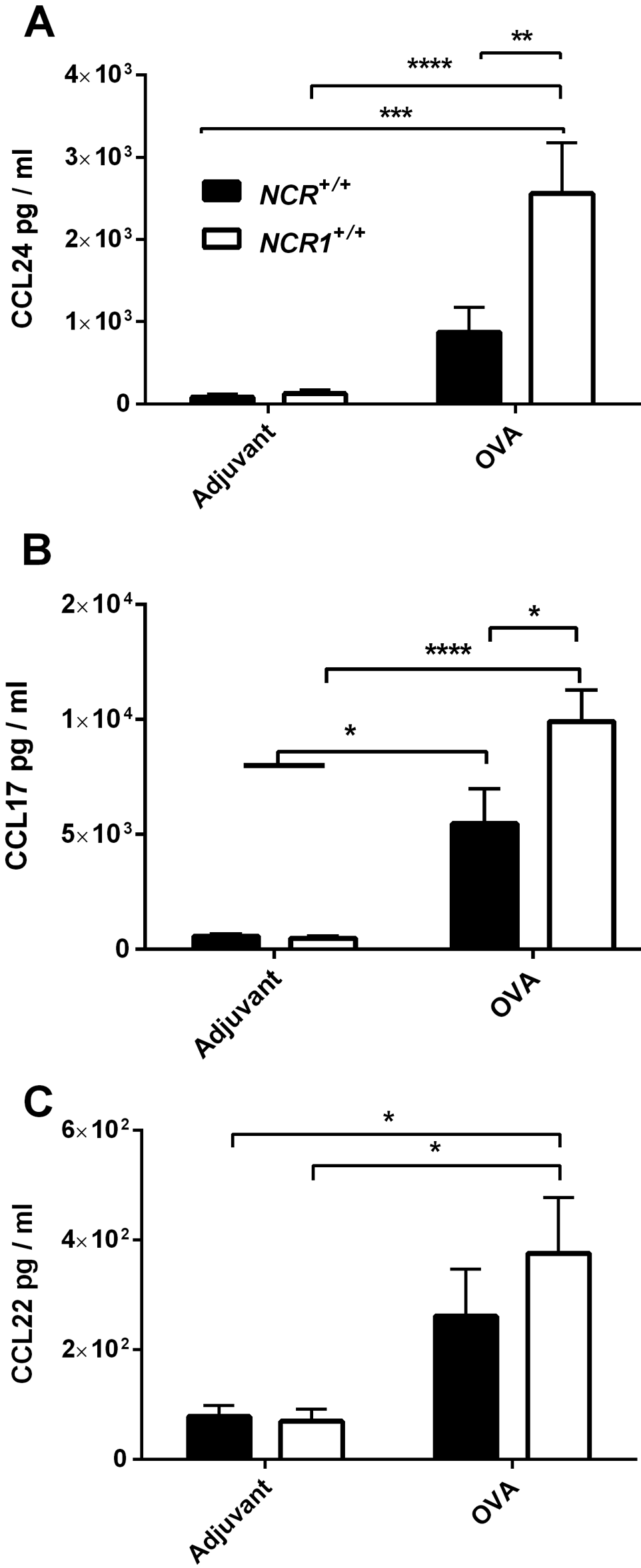

Supplement: S5 Fig — Ncr1+/+ and Ncr1gfp/gfp C57Bl/6 mice were i.p. immunized with either OVA or adjuvant on days 0 and 14. Ten days after the second immunization, mice were challenged twice intranasally with OVA at days 24 and 27. 24 h following the second challenge, BALF was taken from each mouse and used in ELISA to detect levels of (A) CCL24 (n = 8 to 11) and (B) CCL17 (n = 9 to 10) and (C) CCL22 (n = 9–10). **p<0.01, ***p<0.001 (One tail ANOVA multiple comparison). (TIF) [file pone.0160779.s005.tif]
