# Supplementary material for: Natural Killer Receptor 1 Dampens the Development of Allergic Eosinophilic Airway Inflammation
Source: PLoS One. 2016 Aug 31;11(8):e0160779. doi: 10.1371/journal.pone.0160779 (PMC5007051; doi:10.1371/journal.pone.0160779)
Supplement: S1 Table — (DOCX) [file pone.0160779.s006.docx]

**Table S1: Quantitative Real Time primers.**

| **Catalog No** | **Company** | **Reagent** |  | **Primers** | |
| --- | --- | --- | --- | --- | --- |
|  | SIGMA | SYBR Green | CCGCAGAGGTCCAAGTTC | FW | IL-2 |
|  |  |  | TGAGCAGGATGGAGAATTACA | Rev |  |
|  |  |  | ATGTGCCAAACGTCCTCAC | FW | IL-4 |
|  |  |  | CACCTTGGAAGCCCTACAGA | Rev |  |
|  |  |  | CTGCAAGAGACTTCCATCCAGTT | FW | IL-6 |
|  |  |  | GAAGTAGGGAAGGCCGTGG | Rev |  |
|  |  |  | AGACCAGACTCCCCTGTGCA | FW | IL-13 |
|  |  |  | TGGGTCCTGTAGATGGCATTG | Rev |  |
|  |  |  | CAAATGGCCTCCCTCTCAT | FW | TNF alpha |
|  |  |  | CACTTGGTGGTTTGCTACGA | Rev |  |
|  |  |  | TCAAGTGGCATAGATGTGGAAG | FW | IFN gamma |
|  |  |  | TGACGCTTATGTTGTTGCTGA | Rev |  |
| RE00032262-001 |  |  | TGAAATTGTGCTGCTGACCGATGG | FW | Gobs |
| RE00032263-001 |  |  | TGTCTGCAGGCCTCCTGTCATTTT | Rev |  |
| RE00032260-001 |  |  | ACCAACAGCCCCCATCTTTGATGA | FW | Muc5ac |
| RE00032261-001 |  |  | TGCAGATGCAGGAATCGCAGTT | Rev |  |
| RE00050592 |  |  | GCTGGTATAAGACCTCAGTGGAGTGT | FW | CCL17 |
| RE00050953 |  |  | CAATCTGATGGCCTTCTTCACA | Rev |  |
| Mm00439618_m1 | Applied Biosystems (AB) | TaqMan Gene Expression |  |  | IL-17 |
|  |  |  |  |  |  |
| Mm00443258_m1 |  |  |  |  | TNF α |
|  |  |  |  |  |  |
| Mm01168134_m1 |  |  |  |  | IFN γ |
|  |  |  |  |  |  |
|  |  |  | CACCTCACAAGAGGCACAG | FW | IL-17 |
|  |  |  | CAGCAGCAGCAACAGCATC | Rev |  |
|  |  |  | GGTCTCAAACATGATCTGGG | FW | β actin |
|  |  |  | GGGTCAGAAGGATTCCTATG | Rev |  |
|  |  |  |  | FW | *NCR1* |
|  |  |  | CTA GGGCTCACAGAGGGACATAC  CAACACCTCCTGTGATGAGTAGT | Rev |  |
